# Highly mercury-resistant strains from different Colombian Amazon ecosystems affected by artisanal gold mining activities

**DOI:** 10.1007/s00253-022-11860-y

**Published:** 2022-03-28

**Authors:** Gladys Inés Cardona, María Camila Escobar, Alejandro Acosta-González, Patricia Marín, Silvia Marqués

**Affiliations:** 1 Instituto Amazónico de Investigaciones Científicas SINCHI, 110321 Bogotá, Colombia; 2 Faculty of Engineering, Universidad de La Sabana, Chía, Colombia; 3 Consejo Superior de Investigaciones Científicas, Estación Experimental del Zaidín, Department of Environmental Protection, Granada, Spain

**Keywords:** Mercury, Methyl mercury, *merA* gene, *Pseudomonas*, *Bacillus*, *Burkholderia*, Colombian Amazon, Antibiotic resistance

## Abstract

Two sites of the Colombian Amazon region with different levels of human intervention and mercury pollution were selected for the collection of samples of river and lake water, sediments, and associated forest soils. The Tarapacá region, affected mainly by barrage mining, showed low mercury concentrations, whilst in the Taraira region, affected by underground mining, there were several points with high mercury pollution levels. A collection of 72 bacterial and 10 yeast strains with different levels of mercury resistance was isolated and characterized. Most of the highly resistant bacterial strains (MIC > 40 mg L^−1^ HgCl_2_) were isolated from soil and sediment samples and belonged to either *Pseudomonas* (60%) or *Bacillus* (20%). Most of highly resistant bacterial strains were positive for the presence of the *merA* gene, suggesting an active mercury resistance mechanism. This was confirmed in the two most resistant strains, *Pseudomonas* sp. TP30 and *Burkholderia contaminans* TR100 (MIC = 64 and 71 mg L^−1^ HgCl_2_, respectively), which in the presence of increasing mercury concentrations expressed the *merA* gene at increasing levels, concomitant with a significant mercury reduction activity. Analysis of the MerA sequences present in the different isolates suggested a high gene conservation within the taxonomic groups but also several horizontal gene transfer events between taxonomically distant genera. We also observed a positive correspondence between the presence of the *merA* gene and the number of antibiotics to which the strains were resistant to. The most resistant strains are good candidates for future applications in the bioremediation of mercury-contaminated sites in the Amazon.

**Key points**

• *Amazon sediments affected by underground gold mining have higher Hg levels.*

• *Highly Hg-resistant isolates belonged to Pseudomonas and Bacillus genera.*

• *TR100 and TP30 strains showed remediation potential to be used in the Amazon region.*

**Graphical abstract:**

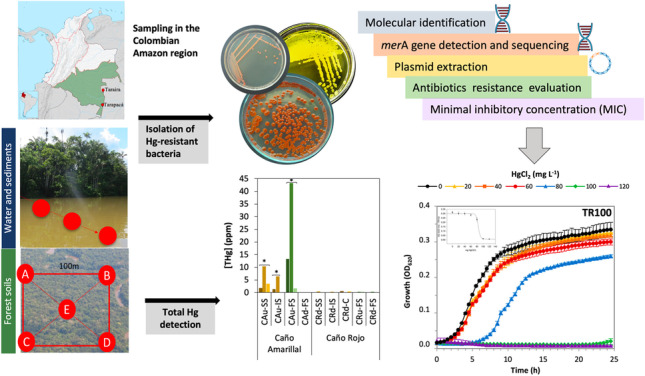

**Supplementary Information:**

The online version contains supplementary material available at 10.1007/s00253-022-11860-y.

## Introduction

One of the major concerns in the Amazon rainforest ecosystem today is the pollution of water, forest soil, and water body sediments with mercury (Hg). Due to the persistence of a long-running armed conflict and the presence of rebels in the area, the inland Amazonian regions of southern Colombia have been largely inaccessible to either environmental authorities or scientists. In recent years, researchers have identified an increase in the presence of small-scale illegal gold miners in the region, who routinely use Hg to form an amalgam with gold to separate it from other minerals (Calao-Ramos et al. 2021). In this process, a small proportion of Hg (10%) amalgamates with gold, and the remaining 90% is directly released into the environment. This activity is intense along certain stretches of the river and releases large amounts of Hg into these environments (Salazar-Camacho et al. 2017), which accumulate in water, sediment, and soil (UNODC 2018). In fact, small-scale gold mining with Hg around the world is thought to produce more than 35% of Hg emissions on a global scale (Stoffersen et al. 2018). The degradation of the Amazon Forest due to human activity, and especially gold mining, has significantly increased the release of Hg into this ecosystem. Atmospheric Hg concentrations in the Amazon region have been linked to gold mining activities (63%), biomass burning (31%), soil dust (4%), and the sea salt aerosol component (2%) (Artaxo et al.

2000). However, the largest Hg reservoir in the region is soil, in which Hg is mainly associated with organic matter and other soil components that play an important role in Hg accumulation (do Valle et al. 2005).

At the artisanal scale, gold is normally mined using two main methods: “pocket mining” and “barrage mining.” Pocket mining involves underground extraction of mineral material via tunnels, followed by grinding and cyanidation or amalgamation of the material to extract the gold, whilst in barrage mining, also known as alluvial gold exploitation, gold material is removed from the riverbed with suction pumps or by panning (artisanal mining). During the process to separate the gold from the rest of the material, large amounts of sediments and soils are treated with elemental Hg (Hg^0^), which establishes strong bonds with gold. The gold–Hg amalgam is then heated to separate the metals (Diringer et al. 2015). In Colombia’s Amazon basin, barrage mining takes place above all on certain stretches of the Taraira river (Taraira region) and Putumayo and Cotuhé rivers (Tarapacá region) (Alcala-Orozco et al. 2019).

Gold mining also impacts on the wellness and health of the indigenous communities. The Hg vapors that enter the atmosphere later return, via rainfall, to aquatic ecosystems. Under anoxic conditions, Hg can be transformed by the prokaryotic community into toxic, water-soluble methylmercury (MeHg), which is highly stable and bioaccumulates through the trophic chain (Barkay and Wagner-Dobler 2005; Liu et al. 2014; Tsz-Ki Tsui et al. 2019; Wu et al. 2019). Artisanal gold miners, their families, and those involved in processing gold are the population most at risk from exposure to elemental Hg through inhalation of evaporated Hg and via the ingestion of MeHg contaminated fish (Legg et al. 2015). In the Colombian Amazon Guainia and Vaupés departments, Calao-Ramos et al. (2021) found a relationship between burning of amalgam, high fish consumption, and highest concentrations of Hg in urine and hair in gold miners. Also, in Taraira and Tarapacá, indigenous communities exceed the international standards of safe Hg concentrations in hair (< 1 ppm) (Salazar-Camacho et al. 2017) with mean Hg concentrations in hair of 34.9 ± 2.4 ppm (Taraira) and 10.6 ± 0.4 ppm (Tarapacá) (Alcala-Orozco et al. 2019; Valdelamar-Villegas and Olivero-Verbel 2020).

Mercury undergoes several chemical and physical transformations as part of its biogeochemical cycle. The three valency states of Hg, metallic (Hg^0^), mercuric (Hg^2+^), and mercurous Hg (Hg_2_^2+^), are in equilibrium in nature through disproportionation reactions (O'Connor et al. 2019). Hg vapors (Hg^0^) in the atmosphere can be oxidized to ionic Hg through the action of ozone in the presence of rainwater, so returning this inorganic Hg to the earth’s surface. The dominant Hg species are Hg(OH) and HgCl_2_ in surface waters, HgS (cinnabar) in sediments, and Hg^0^ (elemental Hg) in the atmosphere (Dash and Das 2012).

In Hg-polluted habitats, microbial activity is the main mechanism of Hg speciation, although Hg can also be toxic to the microbial community. Hg ions can easily interact with the sulfhydryl ligands in proteins, resulting in protein inactivation and loss of cellular functions (Dash and Das 2012). Furthermore, organic Hg compounds such as MeHg can react with DNA causing irreversible damage (Mathema et al. 2011). Some microorganisms have developed mechanisms for Hg detoxification involving specific genes related to tolerance and resistance. Usually, resistance is connected to active Hg response genes for the enzymatic Hg^2+^ reduction to Hg^0^ or methylation to MeHg, as well as demethylation prior to reduction (Das et al. 2016). In contrast, tolerance involves a general metabolic response normally connected to Hg binding to cell wall constituents, pigments and extracellular polysaccharides (biosorption), and precipitation of inorganic Hg complexes such as HgS around the cells (Gadd 2010).

Hg-resistant bacteria are responsible for the three main biological Hg transformations: Hg^2+^ reduction to Hg^0^, Hg^2+^ methylation to MeHg, and MeHg demethylation. The first and last processes are linked to genes in the *mer* operon, widely distributed among bacteria and archaea, which includes the genes for Hg transport and mobilization (*mer*P/*mer*T/*mer*C), *merAB* for Hg demethylation and reduction, and *merR*/*merD* for sensing and regulation (Barkay et al. 2003). On the other hand, methylation is associated with anoxic processes related with sulfate-reducing and iron-reducing bacteria and methanogens that depend on the activity of the products of *hgcA* and *hgcB* genes, coding for the enzyme directly responsible for Hg methylation (Parks et al. 2013). The key gene in the detoxification process is *merA*, coding for a reductase that converts Hg^2+^ ions to volatile Hg^0^. For its part, *merB* codes for an alkylmercury lyase, a key step in the detoxification of organomercury compounds that cleaves MeHg into CH_4_ and Hg^2+^, which is further reduced to Hg^0^ by the product of *merA* (Mathema et al. 2011). The *mer* operon has been found in chromosomes, transposons, and plasmids from virtually all eubacterial groups, as well as in Archaea, isolated from highly diverse environments (Rasmussen et al. 2008; Lapanje et al. 2010; Møller et al. 2011; Boyd and Barkay 2012; François et al. 2012; Freedman et al. 2012; Geesey et al. 2016; Chasanah et al. 2018; Matsui and Endo 2018) and references therein). The Hg metabolic capacities of these resistant strains form the basis of bioremediation strategies for the clean-up of Hg-polluted environments (Mahbub et al. 2017; Bravo et al. 2020).

As part of a global analysis of Hg pollution in the Colombian Amazon rainforest and in order to characterize the potential of the microbial response to this pollutant, we sampled different ecosystems in the region exposed to different levels of Hg contamination and isolated a collection of strains that showed different Hg resistance levels. Two localities, Taraira and Tarapacá, were selected for their intense extractive activities such as prospecting mining processes, artisanal gold mining, illegal logging, exploitation of the aquatic and terrestrial fauna, and deforestation, among others. Taraira and Tarapacá are centers of small-scale illegal gold mining, which is facilitated by the fact that they are transit areas towards the borders with Peru and Brazil (Salazar-Camacho et al. 2017). The aim of this study was to isolate and characterize cultivable Hg-resistant bacteria from ecosystems in two sites in the Colombian Amazon with artisanal gold mining history, to obtain proficient strains for future applications in Hg bioremediation strategies.

## Materials and methods

### Site description and sampling

**Fig. 1:**
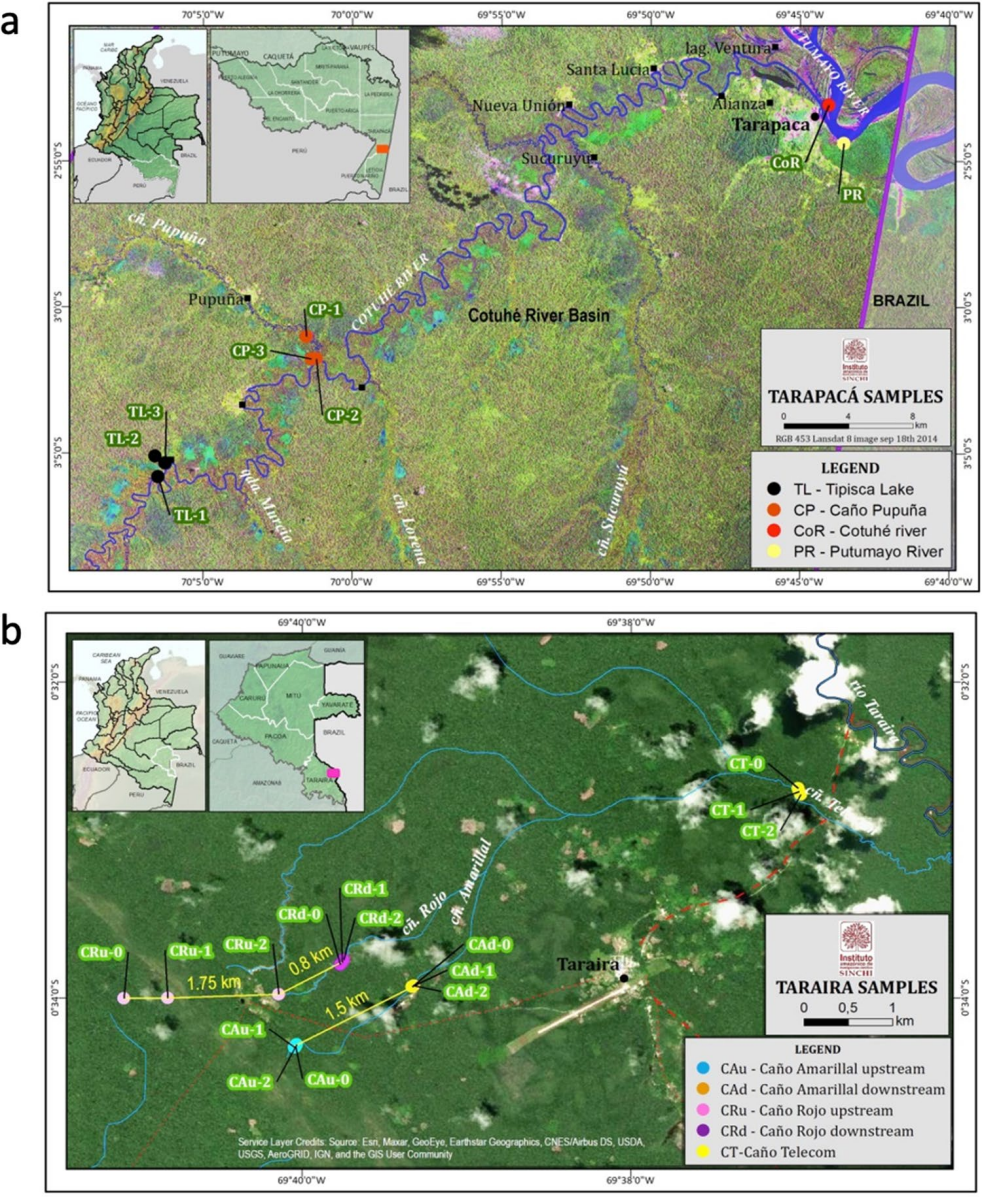
Sampling sites in the two Amazon regions.** a** Map of the Tarapacá region showing the confluence between the Cotuhé and Putumayo rivers. TL, Tipisca Lake; CoR, Cotuhé river; CP, Caño Pupuña; PR, Putumayo River. **b** Map of the Taraira region where the samples were collected. CRu, Caño Rojo upstream stretch; CRd, Caño Rojo downstream stretch; CAu, Caño Amarillal upstream stretch; CAd, Caño Amarillal downstream stretch; CT, Caño Telecom. The names refer to the sampling sites where the water and sediment samples were collected (as indicated in Supplementary Table S1A and B). The forest soil samples, collected in the forest close to the corresponding water sampling site, are not indicated in the maps. The numbers indicate the three replicates collected in each site

Two sites in the Amazon Forest in Colombia were selected for sampling: one was in the Tarapacá area (Amazon Department, Colombia), characterized by barrage gold mining, and the other was in the Taraira municipality (Vaupés Department, Colombia), where gold is extracted by underground mining. Samples were taken during the dry seasons at several sites within each location, as detailed in Supplementary Table S1. The four sampling sites in Tarapacá were connected by the water network of the Cotuhé river system (average flow rate 100 m^3^/s) and included two sites in the Cotuhé river basin (Fig. 1a): (1) the Tipisca lake (TL), a busy mining center due to the intense traffic of gold dredges from the Putumayo river (average flow rate 312 m^3^/s), and (2) the Caño Pupuña creek (CP), part of a sacred site for the local indigenous people which therefore should have minimal anthropogenic pollution. The other two sites in Tarapacá were at the junction between the Cotuhé river and the Putumayo River (CoR) and at the Putumayo River (PR). In the Taraira region, three small creeks were sampled in the Taraira highlands with flow rates of less than 5 m^3^/s: Caño Amarillal (CA), Caño Rojo (CR), and the effluent of both, Caño Telecom (CT) (Fig. 1b). The samples from CA and CR were taken at both the upstream (labelled with u) and downstream (labelled with d) stretch of the creeks, with a distance of 0.8–1.75 km between the two (indicated in Fig. 1). In both locations, samples were collected in June 2016 (Tarapacá) and in October 2016 (Taraira) from the water column (W), from surface (SS), interstitial (IS), and deep sediments (DS), as well as from surrounding forest soils (FS). The deep sediment samples were divided into three layers: superficial (DS-S), intermediate (DS-I), and deep (DS-D) (Supplementary Fig. S2).

#### Water column sampling

Water column samples were collected with a plastic container at 10–20 cm depth in each aquatic ecosystem and stored either in 1 L plastic bottles to be used for microbial diversity and total Hg analysis, or in 500 ml amber glass bottles previously treated with nitric and sulfuric acids, to be used for determining the physicochemical parameters (Supplementary Fig. S1a). In TL, CoR, and PR, the samples were collected at different distances from the shore, as follows: the width of the water body was divided into four hypothetical equal segments and water, and sediment samples were collected at each quarter of the total width. In the CP and the upstream and downstream segments of CA and CR, water and sediment samples were collected at three points along a short stretch of river, with 2 m between each sampling point (Supplementary Fig. S2a). For the microbial analysis, the water (1.5–2 l) was filtered in situ using 0.22 µm pore size membranes (Millipore), which were stored at − 4 °C in 1 ml 0.85% NaCl until use. For the physicochemical analysis, the water samples were acidified with HNO_3_ to pH < 2 and kept refrigerated until analysis. Eight unfiltered water samples were collected in Tarapacá site and fifteen unfiltered water samples in Taraira.

#### Sediment sampling

At each water sampling site, composite superficial sediment samples (representative subsamples of sediment top 5 cm) were collected from a boat using an Eckman-Birge type dredger (Supplementary Fig. S1b). Interstitial sediments were collected by inserting transparent acrylic tubes (15 cm long, 5 cm diameter) into the sediments in the banks of the lake, rivers, and creeks. Deep sediment push-cores were collected using 50-cm length plastic cylinders (7 cm inner diameter) from the center of the lake or smaller rivers. The cores were sliced into 3 sections according to depth: superficial (1–7 cm), medium (8–14 cm), and deep (15–21 cm) (Supplementary Fig. S2c and d). The samples with the superficial, interstitial, and deep sediments were placed in plastic bags, labelled, and packed in ice until they were transported to the laboratory, where they were finally preserved at 4 °C (for physicochemical and microbiological analysis) or − 80 °C (for molecular analysis). Twenty sediment samples were collected in Tarapacá and twenty-six in Taraira.

#### Forest soil sampling

At each sampling site, forest soil samples (taken from the 0–20 cm topsoil layer) were collected from five points of a hypothetical 20 m × 20 m square plot (Supplementary Fig. S3): samples from the four corners were mixed in groups of two samples to obtain two composite replicates, and a third replica was collected at the center of the plot (Navarrete et al. 2015). The plots were located, walking downstream, on the right bank of the TL, the left bank of CP, and the right bank of the upstream and downstream segments of CA, CR, and CT (Fig. 1). The samples were labelled and packed in ice until they were transported to the laboratory, where they were preserved at 4 °C for physicochemical and microbiological analysis and at − 80 °C for molecular analysis. Six soil samples were collected in Tarapacá and fifteen in Taraira.

### Total mercury and MeHg determination in water, sediments, and soils

All Hg analyses were carried out in the Laboratory of Toxicology and Environmental Management at the University of Cordoba (Montería, Colombia), as previously described (Marrugo-Negrete et al. 2008). Samples for total mercury (THg) analysis were digested with H_2_SO_4_-HNO_3_ (7:3, v/v) and KMnO_4_ at 100 °C for 1 h (USEPA 1998), and the THg was determined by cold vapor atomic absorption spectroscopy (CVAAS) using a Thermo Scientific ICE 3000 series spectrometer as described (Marrugo-Negrete et al. 2010). MeHg analysis was performed by gas chromatography with electron capture detection (PNUMA/FAO/OIEA 1987) as described by Pinedo-Hernández et al. (2015). Samples of 2 g of sediment were extracted by 5 mL KOH/CH_3_OH (25%). Five mL of H_2_SO_4_ (4 M, saturated with CuSO_4_), 5 mL of KBr 4 M, and 4 mL of toluene were added. After centrifugation (2200 rpm for 10 min), the supernatant organic phase was collected. The solvent extraction was repeated three times with 2 mL toluene. The collected organic extract (10 mL) was twice to a back-extraction with 1.0 mL of a cysteine solution (1.0%). The two cysteine extracts (1 + 1 mL) were collected and extracted with a mixture of toluene (0.5 mL), CuSO_4_ saturated solution (0.5 mL), and 4 M KBr (1.0 mL. The organic phase was separated from the aqueous phase, and 2.0 µL was injected into a GC_ECD Perkin Elmer, Autosystem XL. The T-Hg estimation was validated with the certified reference material “Estuarine sediment” (IAEA-405, T-Hg 810 ng g^−1^ and MeHg 5.49 ng g^−1^) used to calibrate the instrument. The percentages of recovery for T-Hg and MeHg were 98.2 ± 0.21 and 96.7 ± 0.43, respectively. The detection limits for T-Hg and MeHg were 14 ng g^−1^ and 2.5 ng g^−1^, respectively.

### Isolation of Hg-resistant bacteria

The isolation of bacteria from the different sample types was carried out according to a modified version of the methods proposed in Kargar et al. (2012) and François et al. (2012). For the isolation of mercury-resistant bacteria (MRB), three replicates of each sample were mixed and handled as a single sample. The MRB were isolated using two methods: direct plate inoculation and culture enrichment, as described below. In both methods, colonies with different morphologies were selected based on color, shape, transparency/opacity, elevation, margins, brightness/mate, and any other observable difference after 72 h (direct plating) or 48 h (after the subculturing process) incubation at 30 ºC. All strains isolated in this study are deposited in the Sinchi Institute Culture Collection of microorganisms (COLMIS). The *Pseudomonas* sp. TP30 and *B. contaminans* TR100 are deposited as COLMIS 147 and COLMIS 204, respectively.

### Direct plate inoculation 

For the direct isolation of the MRB present in the samples, sample suspensions were first prepared by adding 5 g of sample (sediment or soil) to 5 ml 0.85 mM NaCl. The suspension was shaken for 1 h at 100 rpm and 30 °C, and 100 μl of the serial dilutions were plated on LB in the presence and absence (control) of 10 mg L^−1^ (36.8 μM) HgCl_2_. Serial dilutions of the water samples in 0.85% NaCl were plated directly. Plates were incubated at 30 ºC for 3 days.

### Preculturing in the presence of Hg

To improve the selection of MRB, 0.1 g of the samples (soil and sediment) or 100 μl of the cell suspension recovered from the filters (obtained from filtering water samples, see the “4” section) was added to 20 ml of liquid LB medium in the presence and absence of 10 mg L^−1^ of HgCl_2_. The cultures were incubated at 30 °C. Every 24 h, 250 μl of each culture was transferred to 20 ml of the same fresh medium, and growth was followed as OD_600_ (Supplementary Fig. S4). Three transfers were performed (PE1, PE2, and PE3) until 30 μl of the PE3 enrichments or serial dilutions when required were plated on LB supplemented or not with 10 mg L^−1^ of HgCl_2_. The colonies growing on 10 mg L^−1^ HgCl_2_ were selected based on their morphology (Giovanella et al. 2016).

### Determination of the Hg resistance level of the isolates

For the selection of relevant MRB, each colony isolated in the presence of HgCl_2_ as described above was initially tested for its Hg resistance level in liquid medium. Each isolate was first grown on 10 ml LB for 48 h, cells were then collected by centrifugation and suspended in 0.85% NaCl, and the volume was adjusted to reach an OD_600_ of 0.4–0.5. To correlate OD_600_ values with colony-forming units (CFU), serial dilutions of the cell suspension were plated on LB. To obtain a preliminary value of Hg resistance, 100 μl of the cell suspensions was inoculated in 5 ml LB in 10 ml tubes supplemented with the following HgCl_2_ concentrations: 0, 5, 10, and 15 mg L^−1^ (0, 18.4, 36.8, and 55 μM, respectively). Cultures were incubated vertically at 30 °C with orbital shaking a 180 rpm, and growth was followed every 24 h as OD_600_ until stationary phase was reached.

For each MRB strain confirmed with the initial approach described above, the minimal inhibitory concentration (MIC) was determined in a Thermo Scientific Multiskan FC. The strains were grown on LB plates for 24 h; the cell biomass was scraped from the surface and suspended in 2 ml of salt solution (0.85% NaCl) and further diluted to reach an OD_600_ between 1.8 and 2.5. The cell suspensions were then diluted to achieve a final OD_600_ of ~0.1, and 50 μl of the diluted cells was added to 250 μl of LB medium supplemented with the different HgCl_2_ concentrations, ranging from 0 to 120 mg L^−1^ (0.44 mM). Triplicate cultures of each condition were incubated in 96 well plates sealed with a polyester film (Axyseal) at 30 °C and basal shaking speed (5 Hz, 15 mm amplitude), and the OD_600_ was recorded 100 times every hour for 24 h or every 30 min. The MIC was defined as the lowest concentration of HgCl_2_ causing no bacterial growth and was statistically estimated using a modified Gompertz function (Lambert and Pearson 2000), applying a nonlinear regression model with a four-parameter logistic curve using SigmaPlot. According to the degree of growth inhibition and the distribution of the MIC values, three ranges of mercury resistance were established, high (MIC ≥ 30 mg/L HgCl_2_), moderate (10 < MIC < 30), and sensitive, MIC ≤ 10 mg/L HgCl_2_ (Supplementary Fig. S5).

### Taxonomic affiliation

#### 16S rRNA gene amplification for bacteria identification

DNA was extracted from cultures grown on LB using the ZRFungal/Bacterial DNA MiniPrepTM kit (ZymoResearch) according to the manufacturer’s instructions and quantified with Nanodrop and Qubit. The primer pair selected to amplify 16S rRNA gene consisted of 63F (5′-CAGGCCTAACACATGCAAGTC-3′) and 1492R (5′-GGTTACCTTGTTACGACTT-3′) primers (Marchesi et al. 1998). In a final volume of 50 μl, the PCR reaction contained 1 × PCR buffer, 2 mM MgCl_2_, 0.2 mM dNTPs, 0.2 μM of each primer, 0.25 μl Taq polymerase (Platinum-Invitrogen), and 1 μl of the appropriate concentration of DNA (1–10 ng/μl). The PCR conditions were initial denaturation at 95 °C for 2 min, followed by 24 cycles of 30 s at 95 °C, 1 min at 55 °C and 2 min at 72 °C, and a final 10 min extension at 72 °C. The PCR reactions were run on 0.8% agarose gels in TAE buffer (40 mM Tris–acetate, 1 mM EDTA, pH 8.0) for 60–90 min at 100 V. PCR products of an appropriate size were isolated from the gel with the NucleoSpin® Gel and PCR Clean-up kits (Macherey–Nagel) according to the manufacturer’s instructions. The resulting products were quantified with Nonodrop or Qubit and sequenced at Sigmol (Genetic Institute, National University, Bogotá, Colombia).

#### ITS1-5.8S rRNA-ITS2 spacer amplification for yeast identification

Yeast strains were grown on LB plates at 30 °C for 48 h. To isolate the DNA, the cell biomass was collected and extracted in ZR BashingBead Lysis Tubes (Zymo Research) containing 750 μl of lysis solution, according to the manufacturer’s instructions. Yeasts were taxonomically assigned based on the internal transcription spacers (ITS) ITS1-5.8S rRNA-ITS2, using the ITS1-ITS4 primer pair (White et al. 1990). In a final volume of 25 μl, the PCR reaction contained 2.5 mM MgCl_2_, 0.3 mM dNTPs, 0.3 μM of each primer, 0.25 μl Taq polymerase (Thermo Scientific), 1 × PCR buffer, and 1 μl of the appropriate concentration of DNA. The amplification conditions were initial denaturation at 95 °C for 5 min, followed by 40 cycles of 1 min at 94 °C, 1 min at 55.5 °C and 2 min at 72 °C, and a final 10 min extension at 72 °C. The PCR reaction products were processed and sequenced as for 16S RNA gene amplification.

### Amplification of the *merA* gene

For *merA* gene amplification, 5 different primer sets were used: A1S-n.F and A5-n.R, giving a product of 285 bp covering amino acid residues 444 to 539 in the pyridine nucleotide disulfide oxidoreductase dimerization domain of MerA) (Ni Chadhain et al. 2006); merAgram-F and merAgram-R (930 bp spanning residues 150 to 460) (Baldi et al. 2012), merAgram + F and merAgram + R (540 bp spanning residues 443 to 623) (Baldi et al. 2012), A1 and A5 (1238 bp spanning residues 129 to 542 of Tn21 *merA*) (Liebert et al. 1997), and merAHF (5′-CGTSAACGTSGGSTGCGTGCCSTCCAAG-3′) and merAHR (5′-CGAGCYTKARSSCYTCGGMCAKSGTCAGGTAGG-3′) (1205 bp spanning residues 134 to 548 of the *merA* gene of High %G + C bacteria) (Wang et al. 2011). The PCR mix contained 1.5 mM MgCl_2_, 0.2 mM dNTPs, 0.5 μM each primer, 0.25 μl Taq polymerase (Kapa), and 10 ng/μl DNA, except for the reaction with merAFA1/RA5 and merAHF/R primer sets, where the primers were added at 0.3 μM. The amplification conditions for the merAgram-F/R set were as already described (Baldi et al. 2012). The reactions for the remaining primer sets were optimized in our lab. The touchdown conditions for the A1S-n.F/A5-n.R set were as follows: an initial denaturation at 95 °C for 4 min, followed by 9 cycles of 40 s at 94 °C, 30 s at 65 ºC with a temperature decrease of 1 °C per cycle, and 30 s at 72 °C, followed by 30 cycles of 40 s at 94 °C, 30 s at 56 °C, and 30 s at 72 °C and a final 10 min extension at 72 °C. Amplification conditions with the merAgram + F/R set were initial denaturation of 2 min at 95 °C, followed by 30 cycles of 30 s at 95 °C, 45 s at 48 °C with a temperature decrease of 2 °C per cycle, and 2 min at 72 °C, followed by a final extension of 10 min at 72 °C. Amplification conditions with merAFA1/RA5 and merAHF/R sets were initial denaturation of 5 min at 95 °C, followed by 30 cycles of 1 min at 95 °C, 1 min at 65.4 °C (merAFA1/RA5 set) or 66 °C (merAHF/R set), and 2 min at 72 °C, followed by a final extension of 10 min at 72 °C. The PCR reaction products were processed and sequenced as for 16S RNA gene amplification.

### Antibiotic susceptibility of Hg-resistant isolates

Antibiotic resistance was determined using the disk diffusion method on LB plates. To prepare the inoculum, each isolate was cultured on liquid LB at 30 °C. Then the culture OD_600_ was adjusted to a value between 1 and 2 and plated homogeneously on the dried surface of a Petri dish (90 mm diameter). Once dried, antibiotic disks were dispensed onto the surface of the inoculated agar plates. The plates were inverted and incubated at 30 °C, and the inhibition zone diameters (IZDs) were measured (mm) after 24 h. Disks of 15 antibiotics were tested, at the indicated concentration (μg/disk). The selected antibiotics were fluoroquinolones, ciprofloxacin (CIP5), norfloxacin (NOR10), and ofloxacin (OFX5); beta-lactams, imipenem (IPM10), piperacillin (PIP100), amoxicillin (AMX25), ampicillin (AM10), and ticarcillin (TIC75); aminoglycosides, kanamycin (KM30), gentamicin (GM10), and streptomycin (S10); macrolides, erythromycin (E15); glycopeptides, vancomycin (VA30); cephalosporine, cefotaxime (CTX30); tetracyclines, tetracycline (TC30); and chloramphenicol (C30). To determine significant differences between the number of antibiotics that negative and positive *merA* strains can resist, a one-way ANOVA was performed with SPSS Statistics 25 software (IBM SPSS, Armonk, NY). The Shapiro–Wilk test was previously used to confirm that the data followed a normal distribution.

### Mercury reduction assay

*Pseudomonas* sp. TP30 and *B. contaminans* TR100 strains were selected to study their Hg reduction capacity. The strains were grown on LB plates for 24 h; the cell biomass was scraped from the surface, suspended in 2 ml of salt solution (0.85% NaCl), and further diluted to reach an OD_600_ between 1.8 and 2.5. The cell suspensions were then diluted to obtain an approximate OD_600_ of 0.1, and 50 μl of the diluted cells was added to 200 μl of LB medium supplemented or not with 40 mg L^−1^ HgCl_2_ in a 96 well microtiter plate and incubated in a Thermo Scientific Multiskan FC at 30ºC and basal shaking (5 Hz, 15 mm amplitude). Assays were run in quintuplicate for each strain and condition. Growth was recorded every 30 min during 24 h. A non-inoculated control (quintuplicate) was run in parallel in the presence and absence of the same concentration of HgCl_2_. The mercury present in each culture and in the control at times 0 and 24 h was measured in 10 μl samples using a DMA-80 Direct Mercury Analyzer (Milestones Srl, Italy). To that end, the 10 ul samples were diluted in a final volume of 2 ml MilliQ water and kept at -20ºC until analysis. The mercury value of the control samples at time 0 was taken as 100%.

### RNA extraction

The *mer*A gene expression was evaluated using reverse transcription quantitative polymerase chain reaction (RT-qPCR). Cultures (5 ml) of *Pseudomonas sp*. TP30 and *B. contaminans* TR100 in LB supplemented with 0, 10, 20, 40, 60, and 80 mg L^−1^ HgCl_2_ (0, 36.8, 73.6, 147, 220, and 294 μM, respectively) were grown for 48 h and collected by centrifugation at 13,000 × g for 5 min. The nucleic acids in the resulting pellets were preserved by adding 1 ml of DNA/RNA Shield (Zymo Research), and pellets were immediately frozen at − 80ºC until RNA extraction. RNA was extracted using Invitrap®Spin Universal RNA Mini Kit (Stratec) under sterile conditions according to the manufacturer’s instructions. The quality of the extracted RNA was checked in agarose gel electrophoresis, and RNA was quantified using the Qubit®RNA HS Assay Kit (Invitrogen). Recovered RNA (1 or 10 ng/ml) was treated with RNase-free DNase I (Bio-Rad iScript™ gDNA Clear cDNA Synthesis Kit) for 5 min at 25ºC to remove any residual DNA, followed by 75 °C for 5 min to inactivate the enzyme. The absence of DNA was subsequently verified by performing PCR with primers (63F/1492R or Eub338F/Eub518R) targeting the 16S rRNA. The synthesis of cDNA was performed with the DNase-treated RNA and the negative reverse transcription control using the iScript™ gDNA Clear cDNA Synthesis Kit (Bio-Rad). The resulting cDNAs (~ 1 ng/ml) were used in RT-qPCR reactions to quantify the *mer*A gene expression.

### *merA* expression quantitation by RT-qPCR

Individual standard quantification curves for *merA* were obtained with the cloned *merA* gene of a highly resistant *Acinetobacter* sp. M1 strain available at the Sinchi Institute using primers A1S-n.F/A5-n.R (Ni Chadhain et al. 2006) to quantify *Burkholderia* sp. TR100 *merA* expression and with the cloned *merA* gene of *Pseudomonas* sp. TP30 using merA30F/merA30R primers designed in this study (5′-GAAGGCATTCTGGAGAGCAC-3′/5′-GGTCCAATAGGGGGTGTCTT-3′) to quantify *Pseudomonas* sp. TP30 *merA* expression. The standard quantification curve of 16S rRNA was performed with the cloned 16S rRNA gene of *Bacillus amyloliquefaciens* using Eub338F/Eub518R primers (Fierer et al. 2005). To clone each gene, the amplified fragments obtained with the corresponding genomic DNA and primer pairs were purified (NucleoSpin® Gel and PCR Clean-up; Macherey–Nagel, Düren, Germany), cloned in the pCR™4-TOPO cloning vector (TOPO cloning kit, Invitrogen) and transformed into TOP10 (Invitrogen) *E. coli* cells. In all cases, the plasmids were extracted (Invisorb® Spin Plasmid Mini Two; Invitek GmbH, Germany) and linearized with SacI (Promega, Madison, WI, USA). Linearized plasmid DNA was quantified with Qubit ™ (Invitrogen). The copy number (CN) of the *mer*A and 16S rRNA genes in the linearized plasmid DNA was calculated using the formula CN = (DNA amount (ng) × 6.022 × 10^23^) / (DNA length (bp) × 1 × 10^9^ × 650). Serial dilutions of the DNA solutions with a known gene copy number were used to set up the PCR conditions (efficiency = 90–105% and *R*^2^ > 0.980) and to obtain the standard quantification curves. Each standard curve was performed in duplicate, with more than 5 points of the standard dilutions. Not template controls (NTC) were considered as the limits of quantification of each gene. Points of the standard curve or samples with less than 3.3 cycles of difference with the NTC were considered negative.

The *mer*A and 16SrRNA gene expression was quantified using CFX96™ Real-Time PCR Detection System (Bio-Rad) with cDNAs of *B. contaminans* TR100 and *Pseudomonas* sp. TP30. RT-qPCR amplification reactions contained in 10 µl a final concentration of 1 × SsoAdvanced™ SYBRGreen Supermix, 0.3 mM (for Eub338F/Eub518R and merA30F/merA30R) or 0.5 mM (A1S-n.F/A5-n.R) of each primer, and 2.5 ml of cDNA. The thermal cycles consisted of an initial denaturation of 98 °C for 2 min, followed by 40 cycles at 98 °C for 5 s and 61.2 °C (A1S-n.F/A5-n.R) or 57.5 °C (merA30F/merA30R) for 40 s (*merA*) or 62.5 °C for 30 s (16S rRNA gene). The process was completed with a melting curve from 65 to 95 °C, with temperature increases of 0.5 °C every 5 s. Absolute copy number obtained from the above standard quantification curves and corrected according to cDNA final concentration of *merA* and 16S rRNA genes was used to calculate *merA* expression as percentages with respect to 16S rRNA expression.

### Plasmid extraction

All *merA* positive strains were tested for the presence of plasmids using the method proposed by Kado and Liu (1981) using the TOL plasmid of *Pseudomonas putida* KT2440 as positive control and the NZYMiniprep kit of NZYTech according to the manufacturer’s instructions. The resulting DNA was run on 0.8% agarose gels in TAE buffer for 60–90 min at 100 V.

### Sequence analysis and deposition

Sequencing of the purified 16S PCR products obtained with primers 63F and 1492R was performed at Sigmol (Genetic Institute, National University, Bogotá, Colombia). The sequences were checked and assembled with the Geneious software (v 8.9) (Biomatters Ltd). The 16S rRNA gene sequences were compared with the sequences in GenBank, EZ Taxon (https://www.ezbiocloud.net) (Yoon et al. 2017), and SILVA-LTF132 (www.arb-silva.de). The closest related sequences, together with one representative sequence from each subgroup (99% sequence similarity), were aligned, and a phylogenetic tree was constructed using the neighbor-joining DNA distance algorithm (Saitou and Nei 1987) using the ARB software. The resulting tree topologies were evaluated by bootstrap analysis (Felsenstein 1985) of neighbor-joining data sets based on 1,000 resamplings. To analyze the evolutionary history of the *merA* gene product of the isolates, the longest generated *merA* PCR products were sequenced with the primers used in the PCR reaction (Supplementary Table S2). The *merA* sequences obtained were then translated and compared with the sequences in the databases using the blastp tool. The evolutionary distances were computed with the MEGA-X software (Kumar et al. 2018) using the maximum composite likelihood method (Tamura et al. 2004) and are expressed in the number of base substitutions per site, as the unit of measurement. Initial tree(s) for the heuristic search were obtained automatically by applying neighbor-join and BioNJ algorithms to a matrix of pairwise distances estimated using the JTT model and then selecting the topology with superior log likelihood value.

The 16S rRNA sequences were deposited in GenBank as MW930775-MW930846; the *merA* gene sequences were deposited under submission MW980451-MW980471.

## Results

### Mercury concentrations in an Amazon ecosystem

**Fig. 2:**
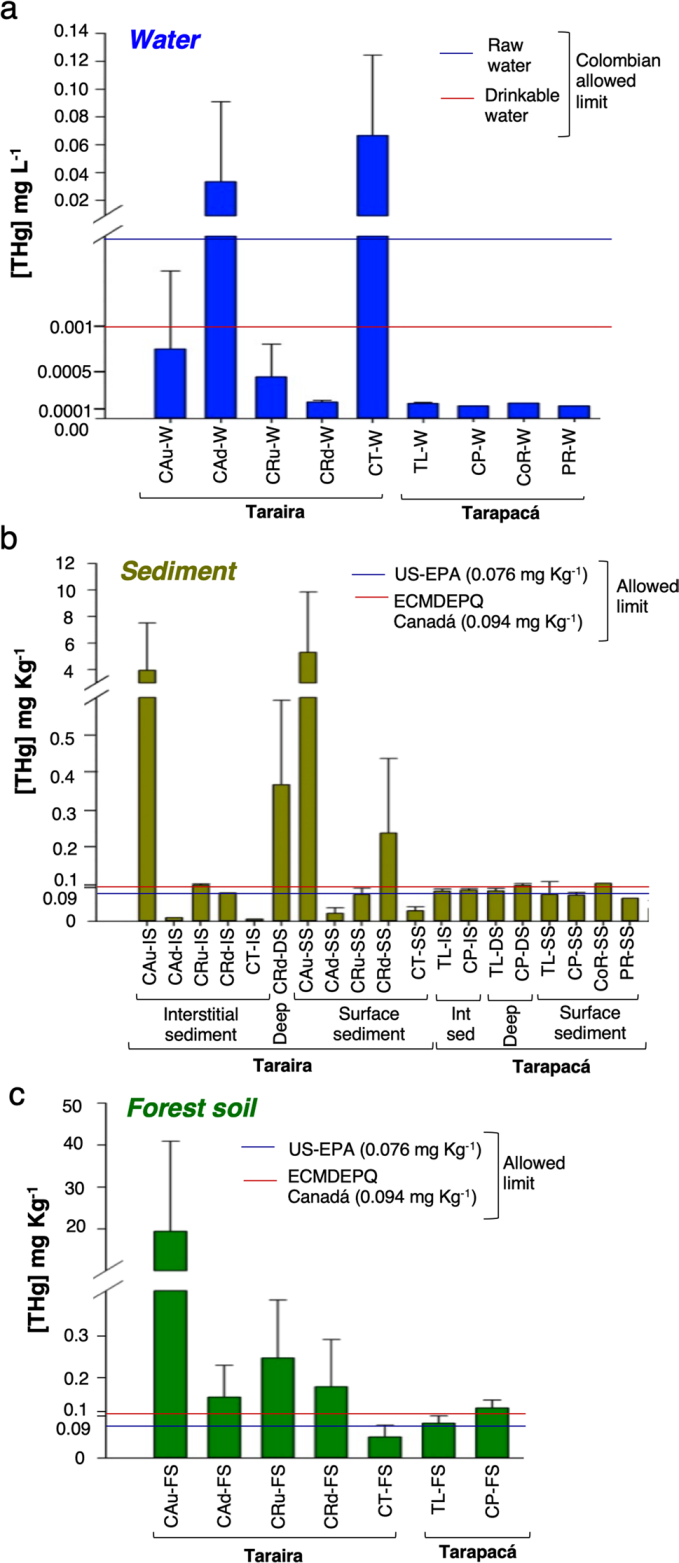
Total Hg concentration in water (**a**), sediment (**b**), and soil (**c**) samples collected from the two locations. Blue and red lines indicate the threshold limit values in each sample type

Water, sediments, and soils from two locations in the Colombian Amazon basin were sampled to characterize the cultivable microbial communities with Hg-resistant capacity in niches influenced by different types of gold mining activities, the Tarapacá area, hit by intensive barrage gold mining, and the Taraira municipality, where underground mining was predominant (Fig. 1, Supplementary Table S1). Mercury (THg and MeHg) was detected in samples from both locations, although overall the pollution levels were significantly higher in the Taraira region than in Tarapacá. In Taraira, THg values clearly exceeded the threshold limit values in almost half (43%) the samples (Fig. 2, Supplementary Table S3). Analysis of the river waters revealed that the highest THg concentrations were found in the samples collected in both the downstream stretch of CA and in CT in the Taraira municipality, reaching 0.07 mg L^−1^ in water samples collected from CT, well above the threshold limit value for water (0.001 and 0.002 mg L^−1^ for drinking and raw water, respectively, according to Colombian regulations) (Fig. 2a). The THg values were significantly higher in the sediment samples and in particular in CA (Fig. 2b), where the highest concentrations reached values that were more than sixty times higher than the international limits for sediments (0.076 mg Kg^−1^ according to USA-EPA and 0.094 mg Kg^−1^ according to Canadian legislation (Bélanger et al. 2007)). The values were not so high in the remaining sediments but were still more than twice as high as the maximum limit in some of the sediment samples from CR. Finally, only the forest soil samples collected in the vicinity of the upstream stretch of Taraira CA showed THg values above the threshold limit (Fig. 2c), so confirming that the region had been severely exposed to THg pollution by artisanal gold mining.

By contrast, the THg values in Tarapacá were below (78% of the samples) or slightly above (22% of the samples) the permitted threshold limit value (Fig. 2, Supplementary Table S3). Only two samples from the Tarapacá area, those collected in the CoR and the CP, reached values that were close to the threshold limit value. All the soil samples had Hg concentration levels below the permitted limits.

The lowest MeHg concentrations were found in the water samples, as expected (Supplementary Table S3). The concentrations of MeHg in the sediments were also quite low, with most of the samples ranging between 0 and 4.85% of the THg present, in accordance with previous analyses of Hg-polluted sediments in the Amazon that established a correlation between the concentrations of THg and MeHg, with an average MeHg/THg ratio of 2.6% (Pinedo-Hernández et al. 2015). The exceptions were the superficial sediment samples of Tipiska Lake (Tarapacá), which showed MeHg values of between 15 and 21.2% of the THg present, suggesting a strong anaerobic microbial activity related to methylation in these sediments. Although they had lower MeHg/THg ratios, the sediment samples from Taraira showed higher MeHg concentrations, and the highest values (0.015–0.034 mg Kg^−1^) were observed in the sites with the highest THg values, i.e., in the upstream stretch of CA (Supplementary Table S3). The highest MeHg concentrations in sediments are consistent with transformation of Hg into MeHg being carried out by anaerobic bacterial groups such as sulfate-reducing bacteria (SRB), iron-reducing bacteria, and methanogens, generally distributed through the sediment profile according to the redox potential (Ma et al. 2019). Hg methylation is generally considered a strategy for Hg detoxification, although recent evidence also suggests that Hg methylation could be a co-metabolic, accidental process (Grégoire et al. 2018).

### Isolation of Hg-resistant microorganisms

**Fig. 3:**
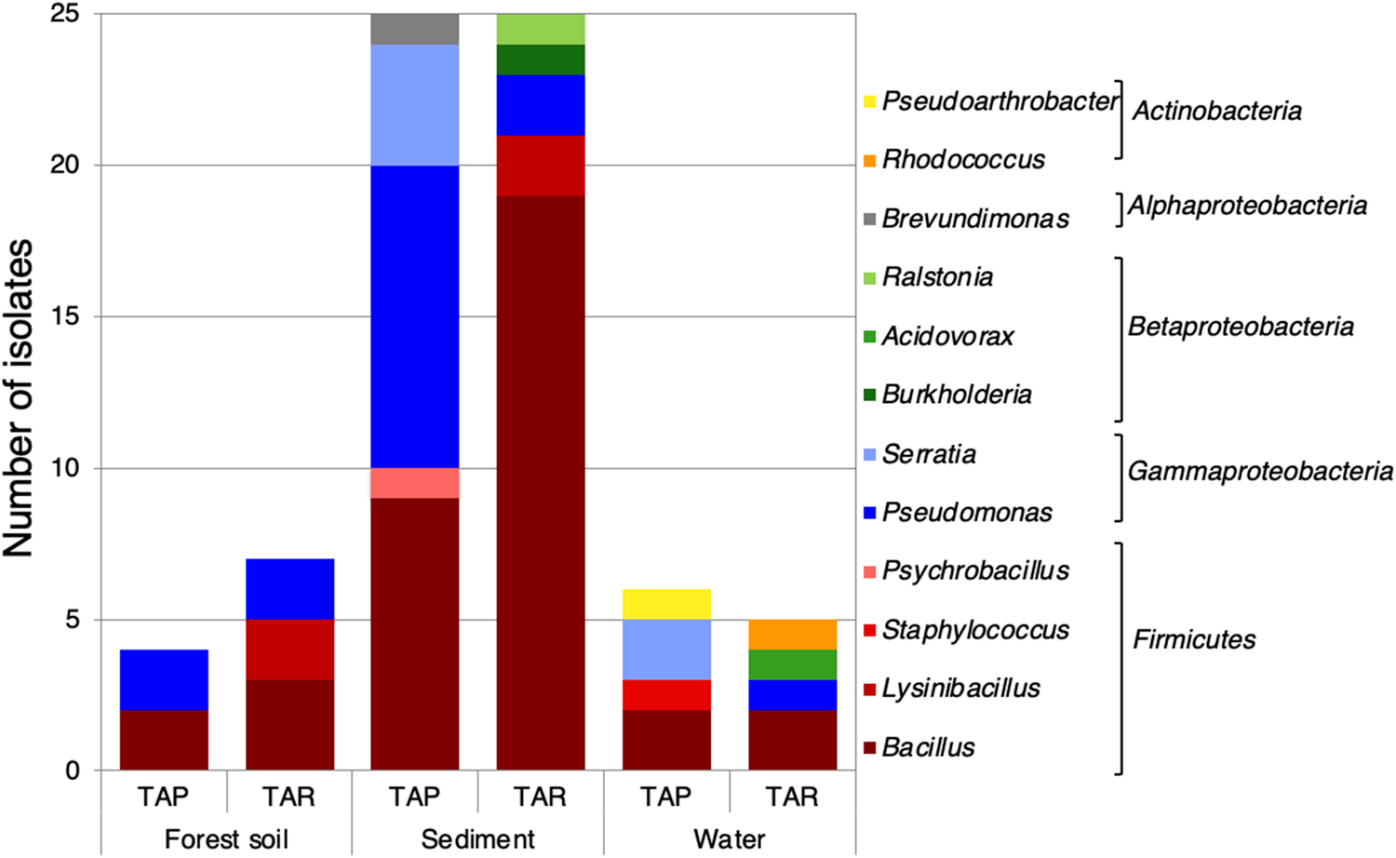
Taxonomic affiliation of the isolates from the two locations. TAP stands for Tarapacá samples; TAR stands for Taraira samples

A total of 72 bacteria and 10 yeast morphotypes which could grow on agar plates at 10 mg L^−1^ HgCl_2_ were isolated from the different samples after a thorough selection and purification process. More than half of the isolates were obtained after direct inoculation of the samples on Hg-containing plates, and the remaining isolates were obtained after three rounds of subculturing on liquid LB supplemented with 10 mg L^−1^ HgCl_2_ (see the 2 section and Supplementary Fig. S4). In general, growth in these cultures in the presence of 10 mg L^−1^ HgCl_2_ was significantly lower than growth in the absence of Hg (Supplementary Fig. S4). The 16S rRNA gene sequence of the bacterial isolates showed that they were mainly affiliated to the phyla *Firmicutes* (55%) and *Proteobacteria* (36%), the latter being especially represented by the class *Gammaproteobacteria*. Figure 3 shows the taxonomic distribution of the strains isolated from each location, and Supplementary Table S4 summarizes their taxonomic affiliation and the environment and location origins. *Gammaproteobacteria* were mainly isolated from Tarapacá samples (51% of the isolates from this location), whilst the strains isolated from Taraira samples were largely represented by the *Firmicutes* (72%). In spite of the small number of MRB isolates obtained from water samples at the TL (Tarapacá), the strains covered a broader taxonomic range. The success of the isolation strategy was greater with sediment samples, probably due to both the higher cell density and the presence of higher levels of Hg in these samples, which favored the selection of Hg-resistant bacteria (Supplementary Table S3A). *Firmicutes* isolates belonged mainly to the *Bacilli*, whilst all the *Gammaproteobacteria* belonged to the genus *Pseudomonas*, except for six *Serratia* strains isolated from Tarapacá samples (Supplementary Fig. S6a and b).

In addition to the bacterial strains, we also identified 10 yeast isolates growing on LB plates with 10 mg L^−1^ HgCl_2._ Seven yeast morphotypes belonged to the *Basidiomycota* and included six *Rhodotorula* strains isolated from different locations and one *Cryptococcus.* The remaining yeast isolates belonged to the *Ascomycota* and included strains of *Aureobasidium*, *Candida* and *Yarrowia* (Supplementary Table S5).

### Assessment of mercury resistance

**Fig. 4:**
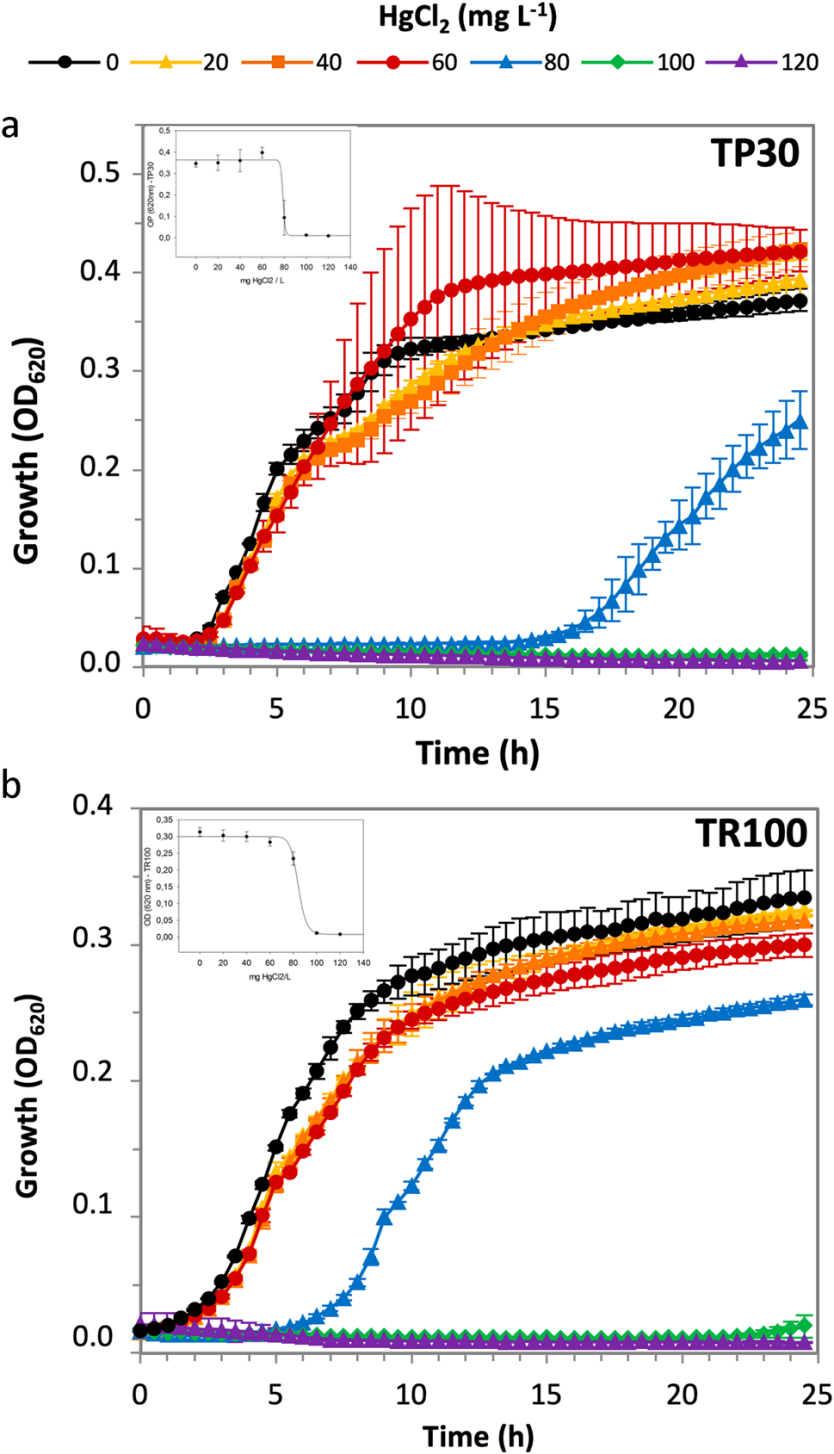
Growth curves of highly Hg-resistant bacteria on different Hg concentrations.** a** *Pseudomonas* sp. TP30; **b** *Burkholderia contaminans* TR100. Over-night cultures on Petri dishes were scraped, resuspended in LB, and used to inoculate 250 µl LB supplemented with 20 (yellow triangles), 40 (orange squares), 60 (red circles), 80 (blue triangles), 100 (green diamonds), and 120 (purple triangles) mg L^−1^ HgCl_2_. Data are the average for two or three separate cultures. The inserts show the MIC calculated with the modified Gompertz function applying a nonlinear regression model with a four-parameter logistic curve

To determine the level of Hg resistance, all Hg-resistant bacteria isolates were assayed for growth in liquid medium supplemented with 5, 10, and 15 mg L^−1^ HgCl_2_. A significant number of isolates (46 strains) that were able to grow in the presence of HgCl_2_ on solid plates were unable to grow with the same HgCl_2_ concentrations in the liquid medium. The strains that managed to resist at least 5 mg L^−1^ HgCl_2_ in liquid medium were selected (30 bacterial and 6 yeast strains) and further characterized by increasing Hg concentrations in microtiter plates, in order to determine Hg minimum inhibitory concentration (MIC). The growth curves in the presence and absence of HgCl_2_ clustered the MRB strains into three categories, according to their Hg resistance level (Table 1). A group of isolates (33%) showed a MIC for HgCl_2_ of over 30 mg L^−1^ (110 μM) and were classified as highly resistant, reaching values close and above 60 mg L^−1^ HgCl_2_ (221 μM). Some of the bacterial strains within this group (TP10, TP13, TP14, TR63) required a lag phase of several hours (10 to 17 h) to initiate growth on 40 mg L^−1^ HgCl_2_ but finally reached OD values similar to those obtained when grown in the absence of Hg. Other strains in the group (TP15, TP16, TR94) could grow at this Hg concentration with or without a reduced (2 h) lag phase (data not shown). At lower Hg concentrations, these strains had no or shorter lag phases. Finally, two strains showed exceptional growth performance in the presence of Hg. *Burkholderia contaminans* TR100, isolated from CR downstream stretch sediment, and *Pseudomonas* sp. TP30, isolated from CP sediment, managed to grow on 20, 40, and 60 mg L^−1^ HgCl_2_ with a similar growth kinetics displayed in the absence of Hg and were also able to grow on 80 mg L^−1^ HgCl_2_ after an initial lag phase (5 h for TR100 and 15 h for TP30), reaching thereafter similar OD values to those obtained when grown in the absence of Hg (Fig. 4). None of the strains could grow in the presence of 100 mg L^−1^ HgCl_2_ and above. A second group of isolates (33%) was categorized as moderately resistant, with a MIC ranging between 10 and 30 mg L^−1^ HgCl_2_. Finally, the remaining isolates (33%) were defined as sensitive because their MIC was below 10 mg L^−1^ HgCl_2_ (36.8 μM) (Table 1).

**Table 1:** Mercury resistance level of the selected isolates

Strain	Taxonomy	Resistance level^1^	Sample origin	MIC (mg L^−1^ HgCl_2_)	*merA*
Bacterial strains					
TP10	*Pseudomonas mohnii*	High	Sediment	52	+
TP13	*Pseudomonas mohnii*	High	Sediment	59	+
TP14	*Pseudomonas mohnii*	High	Sediment	46	+
TP15	*Pseudomonas mohnii*	High	Sediment	58	+
TR16*	*Bacillus toyonensis*	High	Sediment	49	-
TP21*	*Pseudomonas mohnii*	High	Soil	46	+
TP30*	*Pseudomonas* sp.	High	Sediment	79	+
TR63	*Bacillus toyonensis*	High	Soil	44	-
TR94	*Ralstonia insidiosa*	High	Sediment	48	+
TR100	*Burkholderia contaminans*	High	Sediment	86	+
TP19*	*Pseudomonas psychrodurans*	High	Sediment	35	-
TP11	*Bacillus frigoritolerans*	Moderate	Sediment	22	+
TP27*	*Pseudomonas paralactis*	Moderate	Sediment	22	+
TP28*	*Pseudomonas paralactis*	Moderate	Sediment	21	+
TR23*	*Pseudomonas mohnii*	Moderate	Soil	24	+
TR78*	*Bacillus humi*	Moderate	Sediment	23	+
TP33*	*Pseudomonas paralactis*	Moderate	Sediment	11	+
TP45*	*Bacillus nealsonii*	Moderate	Water	12	+
TP3	*Serratia ficaria*	Moderate	Sediment	16	-
TR59	*Pseudomonas paralactis*	Moderate	Water	10.6	+
TR68	*Pseudomonas chlororaphis*	Moderate	Sediment	16.1	+
TR20*	*Bacillus circulans*	Moderate	Sediment	21	+
TP29*	*Pseudomonas paralactis*	Sensitive	Sediment	0.15	+
TP31*	*Bacillus toyonensis*	Sensitive	Sediment	0.16	-
TP32*	*Pseudomonas mohnii*	Sensitive	Soil	0.16	+
TP1	*Serratia marcescens*	Sensitive	Water	7	-
TP34*	*Pseudomonas paralactis*	Sensitive	Sediment	2	+
TP47*	*Staphylococcus petrasii*	Sensitive	Water	(-)^2^	-
TR57	*Acidovorax wautersii*	Sensitive	Water	0	+
TP7	*Serratia marcescens*	Sensitive	Sediment	0	-
TR62	*Bacillus paramycoides*	Sensitive	Soil	0.01	-
TR71	*Bacillus toyonensis*	Sensitive	Sediment	2	-
TR89	*Bacillus simplex*	Sensitive	Sediment	(-)	-
Yeast strains					
TR52	*Rhodotorula mucilaginosa*	High		58	nt
TR55	*Yarrowia lipolytica*	High		51	nt
TR70	*Cryptococcus laurentii*	High		55	nt
TR80	*Rhodotorula mucilaginosa*	High		51	nt
TR58	*Aureobasidium pullulans*	Moderate		24	nt
TR69	*Rhodotorula mucilaginosa*	Sensitive		0.4	nt

^1^ High, MIC ≥ 30; moderate, 10 < MIC < 30; sensitive, MIC ≤ 10

^2^ (-) Data could not be adjusted to the nonlinear model. Visual inspection of the curves classified the strains as sensitive

^*^ Isolated after enrichment on liquid medium with 10 mg L^−1^ HgCl_2_

nt, not tested

All the highly resistant strains were isolated from sediments except for two that came from forest soil samples (TP21 and TR63). Most of the highly resistant strains were isolated from Tarapacá samples and belonged to the *Pseudomonas* genus, whilst the three highly resistant strains isolated from Taraira were more diverse (*Bacillus*, *Ralstonia*, and *Burkholderia*). No highly resistant isolates were obtained from water samples. Approximately half of the isolates, including the two most resistant strains, were obtained after preculturing the corresponding samples on liquid medium supplemented with HgCl_2_ (see the 2 section).

All the resistant yeast strains were obtained from Taraira samples. When tested in microtiter plates as above, two of the *Rhodotorula* strains (TR52 and TR80) and the *Yarrowia* strain (TR55) could also grow at 40 mg L^−1^ HgCl_2_ (147 μM) with no lag phase. Furthermore, the TR80 strain was able to grow on 120 mg L^−1^ HgCl_2_, although it only reached one third of the maximum growth reached in the absence of Hg (not shown). The *Aureobasidium* (TR58) and *Cryptococcus* (TR70) strains showed moderate growth on 40 mg L^−1^ HgCl_2_. The role of yeasts in Hg metabolism is poorly understood. A number of fungi, of which members of *Yarrowia* and *Cryptococcus* genus are among the best characterized, have been reported to tolerate Hg using different mechanisms, through their biosorption capacity, probably mediated by thiol groups on the surface of the cell, or through bioaccumulation via the sequestration of thiol-conjugated Hg to the vacuole (Brunker and Bott 1974; Wysocki and Tamás 2010), with the possible involvement of metallothioneins, phytochelatins, and other cysteine-rich proteins, as a means for SH groups to immobilize Hg in non-toxic form (Durand et al. 2020). Sulfhydryl group-based immobilization is also the mechanism used by several high H_2_S producing isolates, which can co-precipitate Hg in non-toxic form as HgS (Aatif and Zakia 2011). The reduction of Hg^2+^ to volatile Hg^0^ has also been proposed in yeasts (Brunker and Bott 1974; Kelly et al. 2006; Oyetibo et al. 2015), although the potential mechanism involved is unknown.

### Presence and diversity of Hg resistance genes

All the bacterial strains were tested for the presence of the *merA* gene, encoding the detoxifying Hg reductase responsible for the volatilization of Hg^2+^ to Hg^0^. Considering that the *mer*A gene is phylogenetically diverse within and among *Proteobacteria*, *Actinobacteria*, and *Firmicutes* (Boyd and Barkay 2012), different sets of primers were tested against total DNA extracted from bacterial cultures growing on 10 mg L^−1^ HgCl_2_. A *merA* PCR product was obtained in 22 of the initially isolated strains, including all highly and moderately resistant strains except TR63 (Table 1). Interestingly, a *merA* gene was also detected in three strains classified as sensitive, suggesting either that other *mer* genes in the operon were missing or that the presence of a *mer* operon does not guarantee high levels of Hg resistance, and therefore, the involvement of additional factors is required in the resistance mechanism (Leonhäuser et al. 2007). The importance of such additional resistance mechanisms against Hg toxicity in the absence of a *merA* gene is specially suggested in *Bacillus toyonensis* strain TR63, which shows resistance to high levels of HgCl_2_ despite not showing a *merA* copy in its genome.

**Fig. 5:**
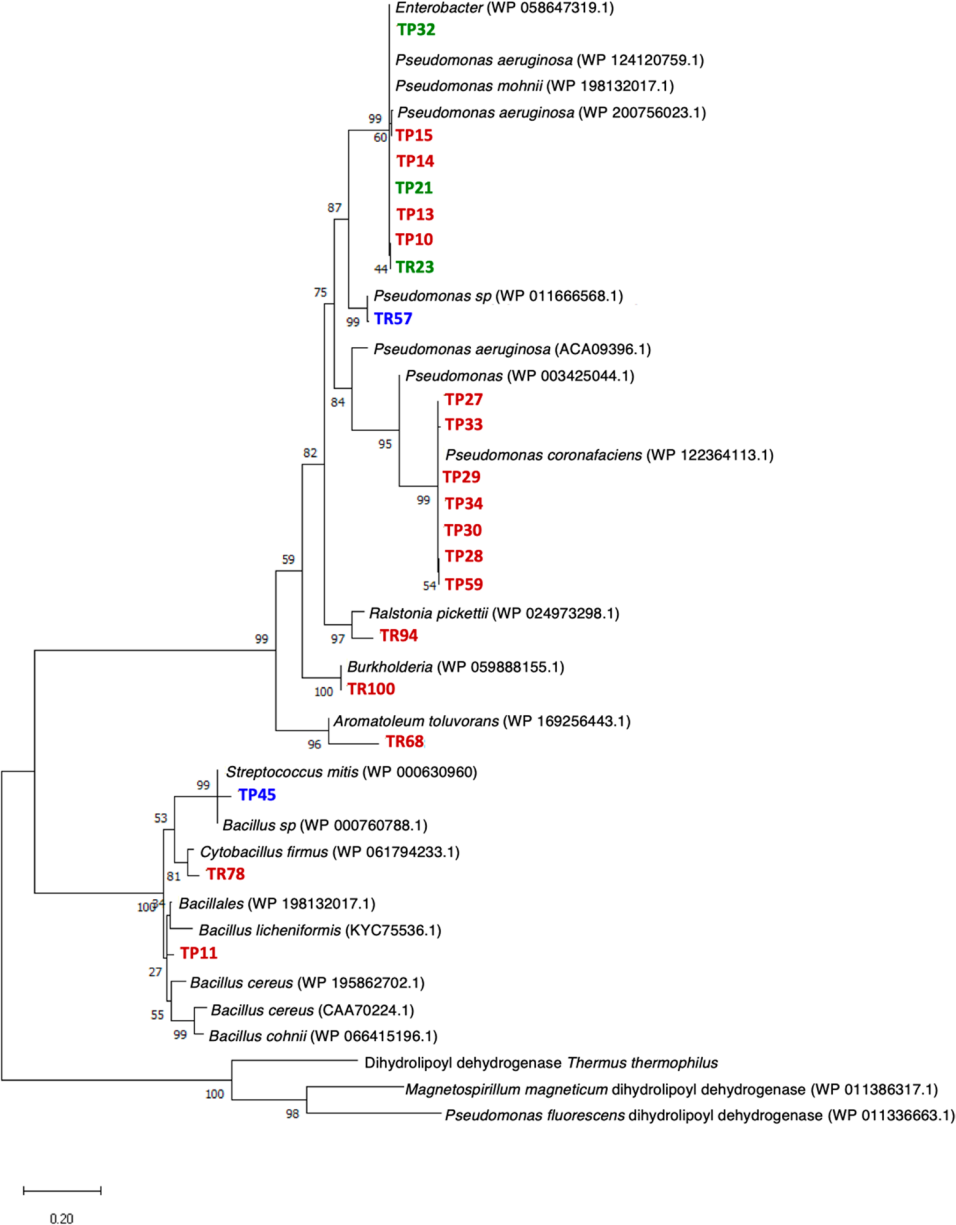
Maximum likelihood phylogeny of the *merA* gene product identified in the isolates. The evolutionary history was inferred by using the maximum likelihood method and JTT matrix-based model (see Materials and methods). The tree with the highest log likelihood (− 12,987.18) is shown. The percentage of trees in which the associated taxa clustered together is shown next to the branches. The tree is drawn to scale, with branch lengths measured in the number of substitutions per site. This analysis involved 43 amino acid sequences. There were a total of 1585 positions in the final dataset. Colors correspond to the isolation origin: water (blue), sediment (red), and forest soil (green). TP, isolated from Tarapacá; TR, isolated from Taraira

The sequences of the PCR products were translated into proteins and compared against protein sequence databases. Figure 5 and Supplementary Table S2 show that most of the closest sequences for some *Gammaproteobacteria* (*Pseudomonas*) and *Betaproteobacteria* isolates belong to sequences reported in species within the same genus (clade of TP27 strain and relatives, strain TR94, and strain TR100) or species from different genera (clade of TP10 strain and relatives, strain TR57, strain TR68). A similar clustering of MerA and 16S rRNA sequences was observed when comparing the isolates within the genus *Pseudomonas*, as observed in both the phylogenetic tree (Supplementary Fig. S6a) and in the MerA protein tree (Fig. 5). The group of MerA sequences clustering with *P. coronafaciens* MerA (TP10, TP13-15, TP21, TR23, TP32) belonged to strains that formed a single OPU related to *P. paralactis* (OPU 4) (Supplementary Table S4), whilst the MerA proteins clustering with *P. aeruginosa* (TP27-30, TP33, TP34, TP59) belonged to strains clustered in a single OPU (OPU 6) close to *P. mohnii* (Fig. 5). Interestingly, although almost all these isolates came from the Tarapacá site, they were obtained from widely differing samples such as those taken from sediments from the TL and CP or from waters from CA in the case of OPU 4 or from sediments from the CoR and soils from the TL, CR, CP, and CA in the case of OPU 6. This suggests that these merA positive isolates represent a widespread *Pseudomonas* lineage in the Tarapacá region. Overall, these observations suggest that horizontal transfer from taxonomically distant organisms had recurrently occurred in these ecosystems. On the other hand, blast results of sequences found in the *Bacillaceae* isolates showed more hits belonging to distant MerA sequences within the same family but not form distant taxa, as observed in *Proteobacteria.*

### Presence of plasmids and antibiotic resistance

It has long been known that Hg-resistant genes are frequently found in association with antibiotic resistance genes in the same mobile elements, such as plasmids and transposons (Wireman et al. 1997), which contribute to the dissemination through lateral transfer of these properties in the environment (Li et al. 2017). To test the presence of antibiotic-resistant genes in the isolates, we assessed the possible resistance of the strains to a set of antibiotics and evaluated the presence of plasmids in the cells (Supplementary Table S6). When we analyzed the simultaneous presence of the *merA* gene and the resistance to different antibiotics, we found that the number of antibiotics that the strains were resistant to was significantly higher in strains bearing the *merA* gene in their genome (*p* = 0.041) (Supplementary Fig. S7), suggesting a high resistance to antibiotics associated to the presence of *merA* and of antibiotic-resistant genes. This was further supported by the observation of the presence of plasmids in 67% of the Tarapacá isolates bearing a *merA* gene, although plasmids were much rarer in the *merA* bearing strains from Taraira (appearing in just one, the highly resistant TR100 strain). However, there was no significant difference in terms of antibiotic resistance between the strains that contained plasmids and those that did not (*p* = 0.839).

### Hg reduction in the highly resistant *Pseudomonas* sp. TP30 and *B*. *contaminans* TR100 strains

**Fig. 6:**
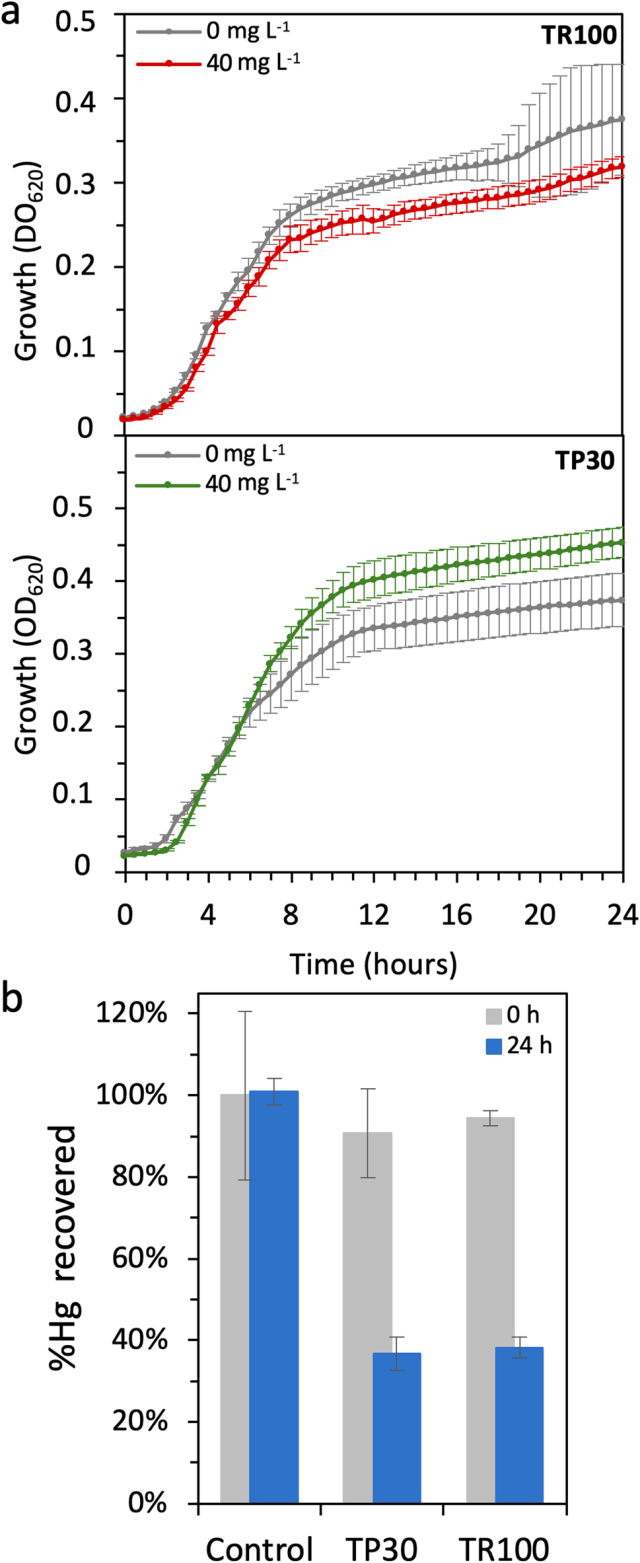
Hg reduction in *Pseudomonas* sp. TP30 and *Burkholderia contaminans* TR100. **a** Growth of *Pseudomonas* sp*.* TP30 and *B. contaminans* TR100 in the presence and absence of 40 mg L^−1^ HgCl_2_. **b** Percentage of Hg recovered from the cultures at time 0 (gray bars) and after 24 h growth (blue bars). An abiotic control of non-inoculated medium in the presence and absence of mercury was included. Experiments were done in triplicate

The highly resistant *Pseudomonas* sp. TP30 and *B. contaminans* TR100 strains were selected to analyze their Hg reduction activity. The two strains were grown on LB containing 0 and 40 mg L^−1^ (147 μM) HgCl_2_ in microtiter plates. Growth of *B.* *contaminans* TR100 was minimally affected by the presence of Hg, whilst growth of *Pseudomonas* sp. TP30 reached OD values slightly higher in the presence of 40 mg L^−1^ of HgCl_2_ (Fig. 6a), as previously observed (Fig. 4a). As abiotic control to estimate Hg volatilization, non-inoculated cultures were included in the assay. At time 0 and after 24 h growth, 100 μl samples were collected, and the concentration of THg was determined. Figure 6b shows that in the abiotic control, 100% of the mercury content was recovered after 24 h, suggesting mercury volatilization was negligible in these conditions. In contrast, in *B. contaminans* TR100 and *Pseudomonas* sp. TP30 cultures. the mercury recovered at time 0 was close to 90%, whilst after 24 h, less than 40% of the initial mercury was recovered. The difference between the percentage of Hg recovered at 0 and 24 h was significant for both strains (ANOVA, *p* = 0.001 for TP30 and *p* = 0.000 for TR100). This indicated a net Hg removal of 50% in both strains, consistent with *merA*-dependent Hg reduction activity.

**Fig. 7:**
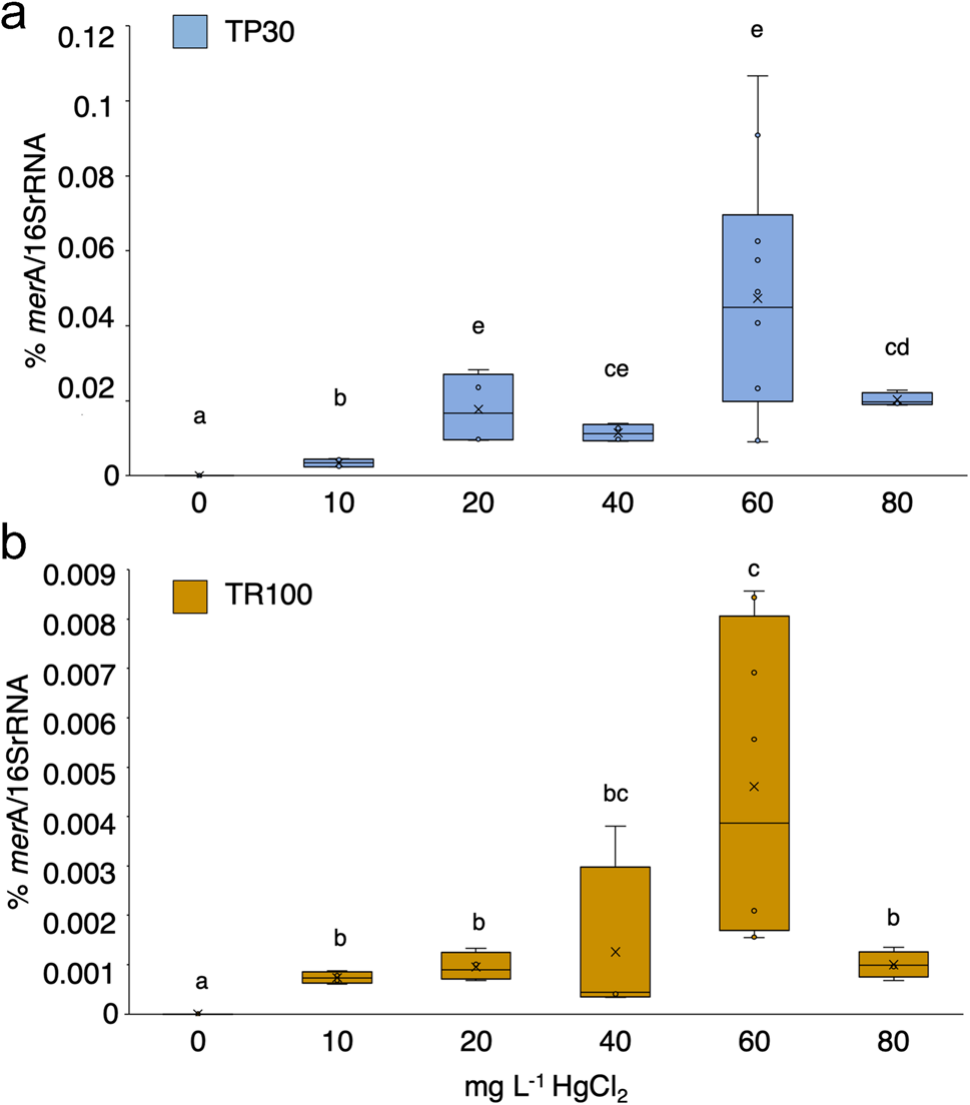
Induction of the *merA* gene expression in *Pseudomonas* sp. TP30 and *Burkholderia contaminans* TR100. Cultures of the two strains (5 ml) in LB supplemented with the indicated concentration of mg L^−1^ HgCl_2_ were grown for 48 h, when *merA* gene expression was determined by RT-qPCR as percentage of 16S rRNA gene expression. Letters above the boxes indicate subgroups (**a** < **b** < **c** < **d** < **e**) according to the differences between their means. Dots denote each replica

We further investigated the expression of *merA* in both strains in the presence of increasing concentrations of HgCl_2_ using RT-qPCR. Figure 7 shows that *merA* messenger was undetectable in the absence of Hg, consistent with the strong MerR-dependent repression of *mer* operon expression generally observed in most *mer* operons (Barkay et al. 2003). Expression of *merA* was readily induced in the presence of 10 mg/L Hg (36.8 μM) and increased with increasing concentrations of Hg in the medium, although the variability of the results was high. Both strains bear an active *mer* operon, which probably reduces the actual intracellular mercury concentration to which MerR is exposed. Expression was highest at 60 mg L^−1^ HgCl_2_ and decayed thereafter. Interestingly, induction levels were almost one order of magnitude higher in the highly resistant TR30 stain.

## Discussion

The two Amazon Forest locations analyzed in this study had a long history of small-scale gold mining activity with the resulting Hg pollution and were selected as a potential niche for the isolation of Hg-resistant bacteria. The Hg concentrations found in the samples were similar to those observed in previous studies of Amazon soils and sediments from other gold mining areas (Diringer et al. 2015). The samples from Taraira showed the highest Hg concentration values, especially those taken from river sediments and forest soils. Whilst the sampled rivers in the Taraira region were small creeks with relatively slow flows, the Cotuhé river system sampled in Tarapacá was an immense waterbed with a high flow rate, which probably drags spilled Hg to sites further downstream, not sampled in this study. Furthermore, mining-related deforestation was significantly more intense in Taraira than in Tarapacá. Deforestation is known to produce a higher sediment load in waterbeds, causing higher Hg accumulations in the water system (Lino et al. 2019). We found Hg contamination levels well above threshold limits in several samples from the Taraira region. The highest Hg values in Tarapacá were observed above all in sediments collected in TL and CP. These findings were confirmed by the Hg content of the fish caught in these waters, in Taraira between 0.0955 and 1.0439 mg Kg^−1^ THg in fish from the Vaupés River, where the CA, CT, and CR streams flow into and between 0.020 and 2.996 mg Kg^−1^ THg in fish from the TL in Tarapacá (Sinchi Institute report 2019).

The bacterial isolates obtained in this study showed different levels of Hg resistance. The number of strains selected for their Hg resistance was higher in samples from Tarapacá despite the higher Hg values found in Taraira. This is probably due to the specific characteristics of the ecosystems in Tarapacá, which are rich in organic matter, which probably boosted the abundance of bacterial communities, in general at least one order of magnitude higher than in Taraira (not shown). Despite the broad spectrum of ecosystems selected as sources for strain isolation (water from different water body types, different layers of sediments, and different soils), the overall retrieved cultivable diversity was relatively low. The strains finally selected as moderately to highly Hg-resistant belonged to the genera *Pseudomonas* (11 strains), *Bacillus* (3 strains), *Ralstonia*, and *Burkholderia* (one strain each). Most of these strains were isolated from Tarapacá sediments and soils. The *Bacillus* and *Pseudomonas* genera have been frequently associated with gold mining and heavy metal rich environments (Chellaiah and Omine 2012; Liu et al. 2013; Ghosh et al. 2015; Egamberdieva 2016; Oladipo et al. 2018). Furthermore, these two genera appear as dominant cultivable Hg-resistant strains in different environments with diverse communities, showing MIC values in the range of those obtained in this study (Supplementary Table S7). *Bacillus* strains were found to be an important group of Hg-resistant bacteria isolated from ecosystems as diverse as river sediments (Figueiredo et al. 2016), brackish and marine sediments (Narita et al. 2003; Kannan and Krishnamoorthy 2006; Pepi et al. 2011), marine sponges (Santos-Gandelman et al. 2014), and isopod guts (Lapanje et al. 2010), whilst *Pseudomonas* strains predominated in the cultivable fraction of Hg-resistant bacteria in a number of polluted soils (Cabral et al. 2013; Giovanella et al. 2016), Arctic samples (Møller et al. 2011), and isopod guts (Lapanje et al. 2010), among others. The common prevalence of these two genera among the cultivable Hg-resistant communities may also reflect a general bias in the cultivation methods used. It is worth noting that in most of the isolation studies mentioned above, different types of nutrient rich agar media were used, which probably influenced how the recovered cultivable community was selected. However, it has recently been suggested that transposon-mediated *mer* operon horizontal transfer may explain the worldwide prevalence of Hg-resistant bacteria belonging to *Bacillus* (Matsui and Endo 2018). In our study, all the selected Hg-resistant *Bacillus* strains except one (*B. toyonensis* strain TR63) tested positive for the presence of the *merA* gene. TR63 was isolated from the forest soil in Taraira, which showed the highest THg values detected in this study (45 mg Kg^−1^ in the most contaminated sample). *Bacillus* species can produce endospores under stress conditions such as those imposed by Hg pollution, which would help them survive in these highly polluted ecosystems. The high resistance of this strain may also be due to its Hg biosorption and accumulation capacity, as has been described for a number of strains within this genus (François et al. 2012; De et al. 2014; Ekyastuti and Setyawati 2015).

The linkage between Hg- and antibiotic-resistant genes appears to be connected to the presence of integron-associated integrases in the vicinity of the *mer* operon present in certain transposon families, which can integrate a number of antibiotic-resistant genes. They occur together both in plasmids and chromosomes (Pal et al. 2017). We found that the number of antibiotics to which each strain was resistant to was higher in *merA* positive strains (Supplementary Fig. S7 and Supplementary Table S6), although no correlation with the presence of plasmids was observed. This is consistent with the results of a broad analysis of genomes in the databases showing that the presence of metal/biocide-resistant genes was more frequent in chromosomes (15%) than in plasmids (1.2%) retrieved from polluted environments, where the co-occurrence of these metal-resistant genes with antibiotic-resistant genes was detected (Pal et al. 2015). Hg-resistant linkage to mobilizable elements such as transposons, and especially transposons related to the Tn21 family, appears to be an ancient characteristic (at least 8,000 years old). Its links with antibiotic resistance only which appeared in the “post-antibiotic era” and seem to have colonized all types of niches (Pal et al. 2017). Furthermore, transposons can be integrated in plasmids, so increasing their mobilization potential (Hall et al. 2015). Although we did not investigate the possible presence of transposons in our isolates, we did observe a high identity between retrieved *merA* sequences of the isolates belonging to the same taxonomic group. The low frequency of *merA* gene transfer between the genetically distant strains observed (Supplementary Table S2) suggests that although horizontal gene transfer played an important role in the dispersal of the *mer* operon in these Amazonian microbial communities, genetic barriers might be limiting gene transfer between taxonomically distant groups.

Two strains, *Pseudomonas* sp. TP30, isolated from superficial sediments in CP (Tarapacá), and *Burkholderia contaminans* TR100, isolated from CR interstitial sediments (Taraira), showed higher resistance than the remaining isolates. The characterization of the Hg transformation capacities of these strains showed they strongly induced the *merA* operon in the presence of Hg in a manner that was dependent on the metal concentration up to a best-tolerated level (60 mg L^−1^ HgCl_2_); induction of *merA* resulted in a significant Hg reduction in the cultures. The potential of these promising strains in remediation strategies applicable to the Amazon Forest region is currently under evaluation.

## Supplementary Information

Below is the link to the electronic supplementary material.

Supplementary file1 (PDF 1980 KB)

## Data Availability

The datasets used and/or analyzed during the current study are available from the corresponding author on reasonable request.
