# Supplementary material for: Highly mercury-resistant strains from different Colombian Amazon ecosystems affected by artisanal gold mining activities
Source: Appl Microbiol Biotechnol. 2022 Mar 28;106(7):2775–93. doi: 10.1007/s00253-022-11860-y (PMC8990959; doi:10.1007/s00253-022-11860-y)
Supplement: Supplementary file 1 — Supplementary file1 (PDF 1980 KB) [file 253_2022_11860_MOESM1_ESM.pdf]

Applied Microbiology and Biotechnology

SUPPLEMENTARY MATERIAL FOR

Highly mercury resistant strains from different Colombian Amazon ecosystems affected by artisanal gold mining activities

Gladys Inés Cardona[1*]. María Camila Escobar[1]. Alejandro Acosta-González[2], Patricia Marín[3] & Silvia Marqués[3]

[1] Instituto Amazónico de Investigaciones Científicas SINCHI. Bogotá. Colombia

[2] Faculty of Engineering. Universidad de la Sabana. Chía. Colombia

[3] Consejo Superior de Investigaciones Científicas. Estación Experimental del Zaidín. Department of Environmental Protection. Granada. Spain

This file contains 7 supplementary figures and 7 supplementary tables

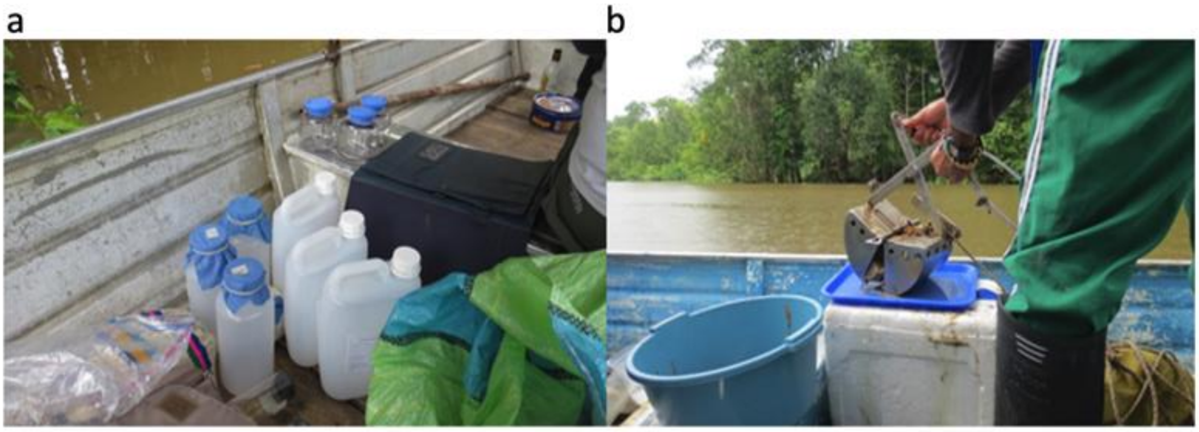

Supplementary Fig S1 a and b. Sampling in the Tipisca Lake (Tarapacá); a) water samples; b) sediment sampled with an Eckman-Birge type dredger.

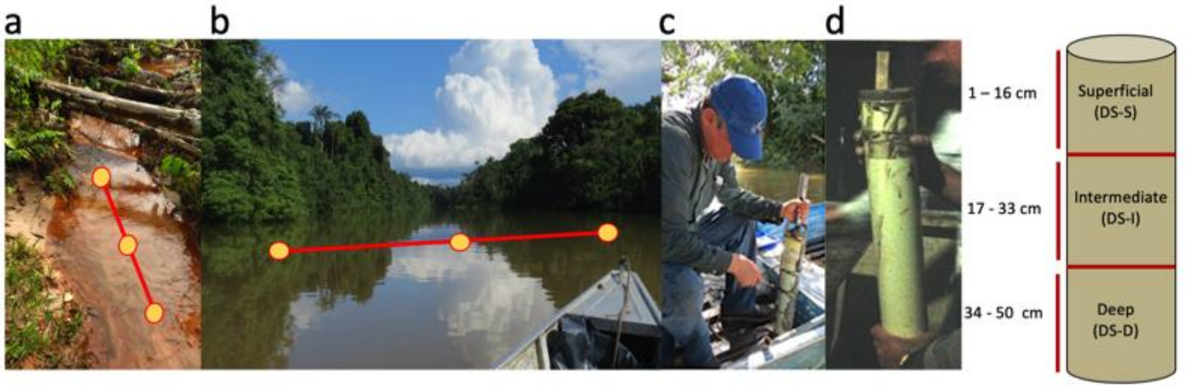

Supplementary Fig S2 a, b, c, and d. River water and sediment sampling in Taraira and Tarapacá. a) Location of the three replicates collected in the mouth of Cotuhé river (Tarapacá). b) Location of the three replicates collected in Caño Rojo creek. c) Deep sediment core collected in the middle of the river. d) Partition of the three layers of the deep sediment cores.

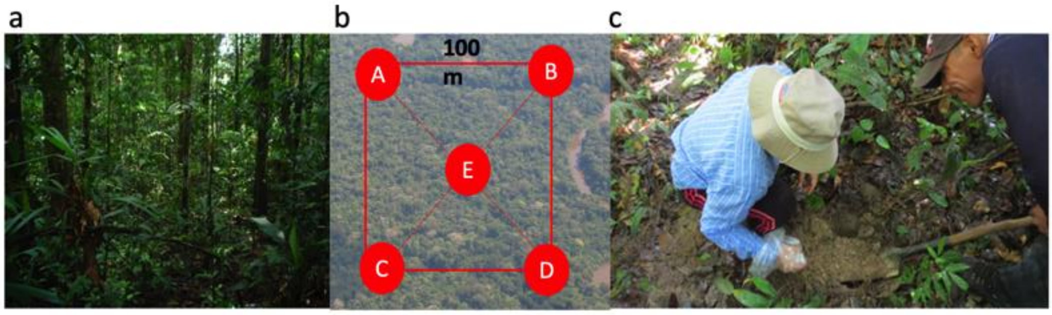

Supplementary Fig S3 a, b and c. a) View of the Amazon Forest in the Taraira region. b) Scheme of the sampling strategy of soil samples. The three replicates consisted of mixed sample pairs A+B, C+D and the single sample E. c) Collecting soil samples in Tarapacá Amazon Forest.

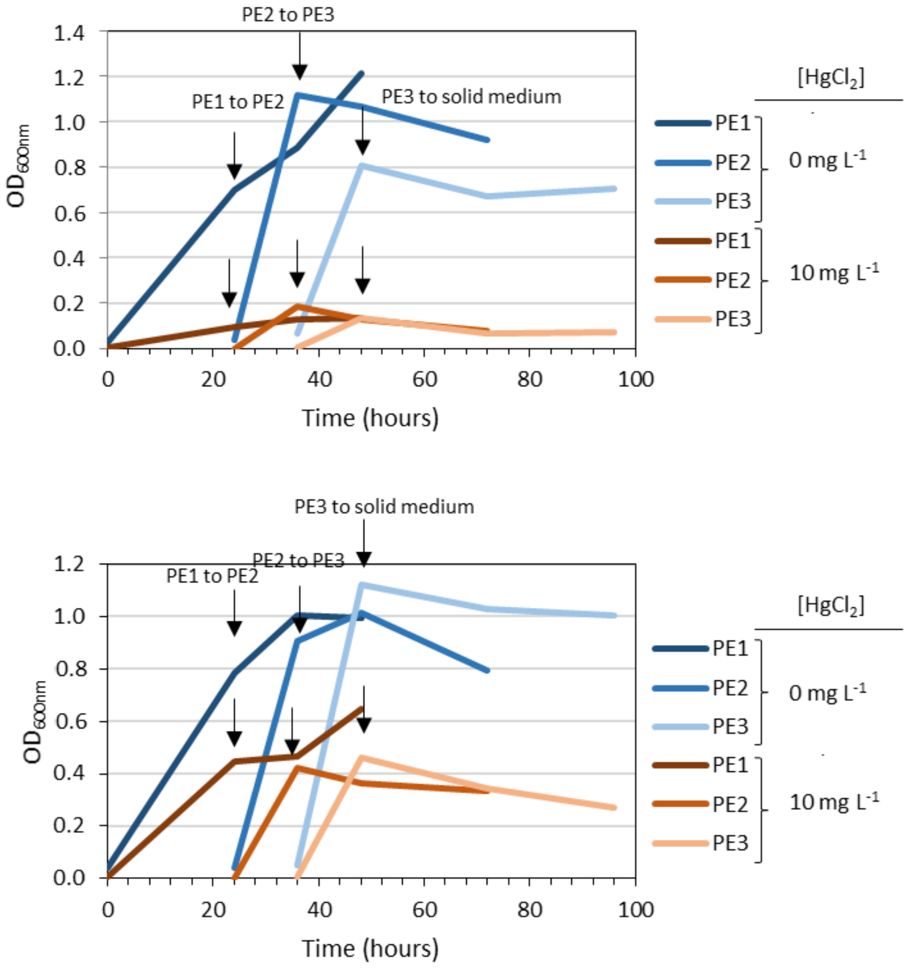

Supplementary Figure S4. Examples of growth of the enrichment cultures.

a) Enrichment from sediment samples collected in the upstream stretch of Caño Amarillal. b) Enrichment from sediment samples collected in the upstream stretch of Caño Rojo. The arrows indicate the dilution of the cultures (PE1, PE2 and PE3) in fresh medium with (brown) and without (blue) 10 mg $HgCl_2$ L^{-1}. The growth curves of parallel cultures in the absence of $HgCl_2$ are shown as control.

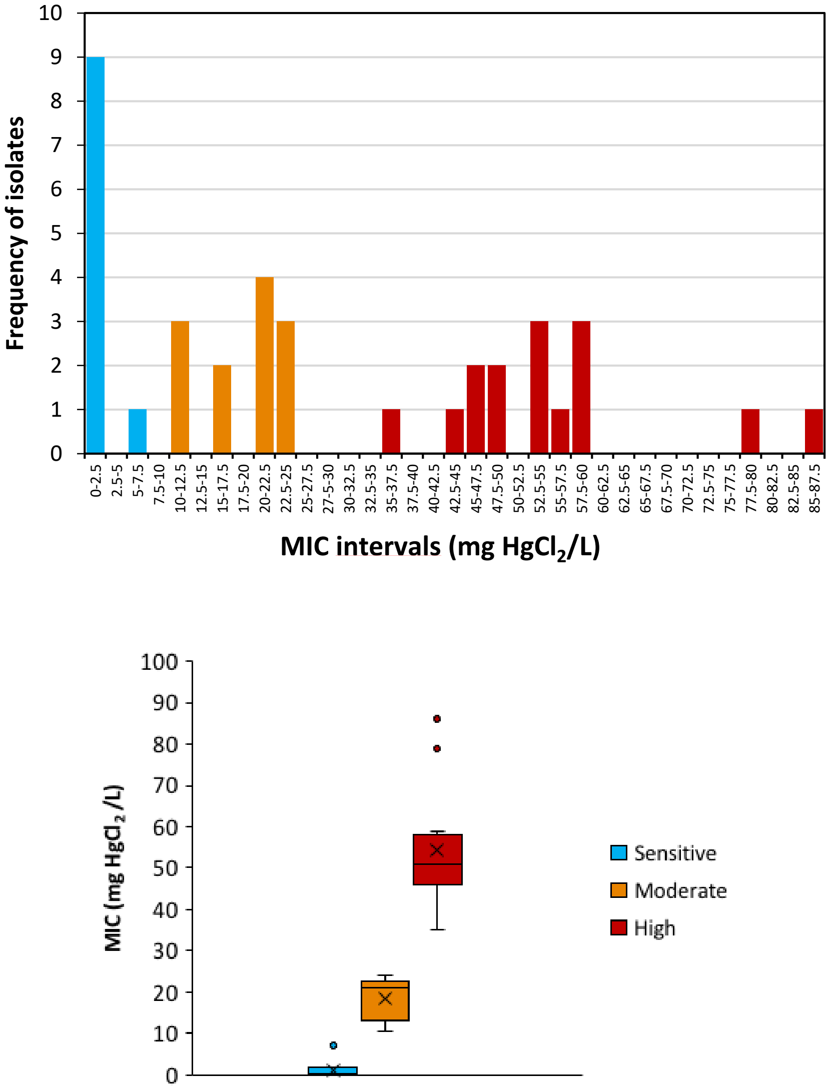

Supplementary Figure S5. a) Distribution of the resistance level of the isolates according to the MIC values determined in this study. The three defined classes are depicted in different colors: blue (sensitive), orange (moderately resistant) and red (highly resistant). MIC values were grouped in intervals of 2.5 mg/L $HgCl_2$. b) MIC box-plot of the defined classes shows significant differences among them (Kruskal-Wallis: p-value = 1.3 x 10^{-07} and Kruskal-Wallis multiple comparison p-values adjusted with the Bonferroni method for High – Moderate: p= 0.001; High – Sensitive: p=2.9e-08 and Moderate – Sensitive: p= 0.02)

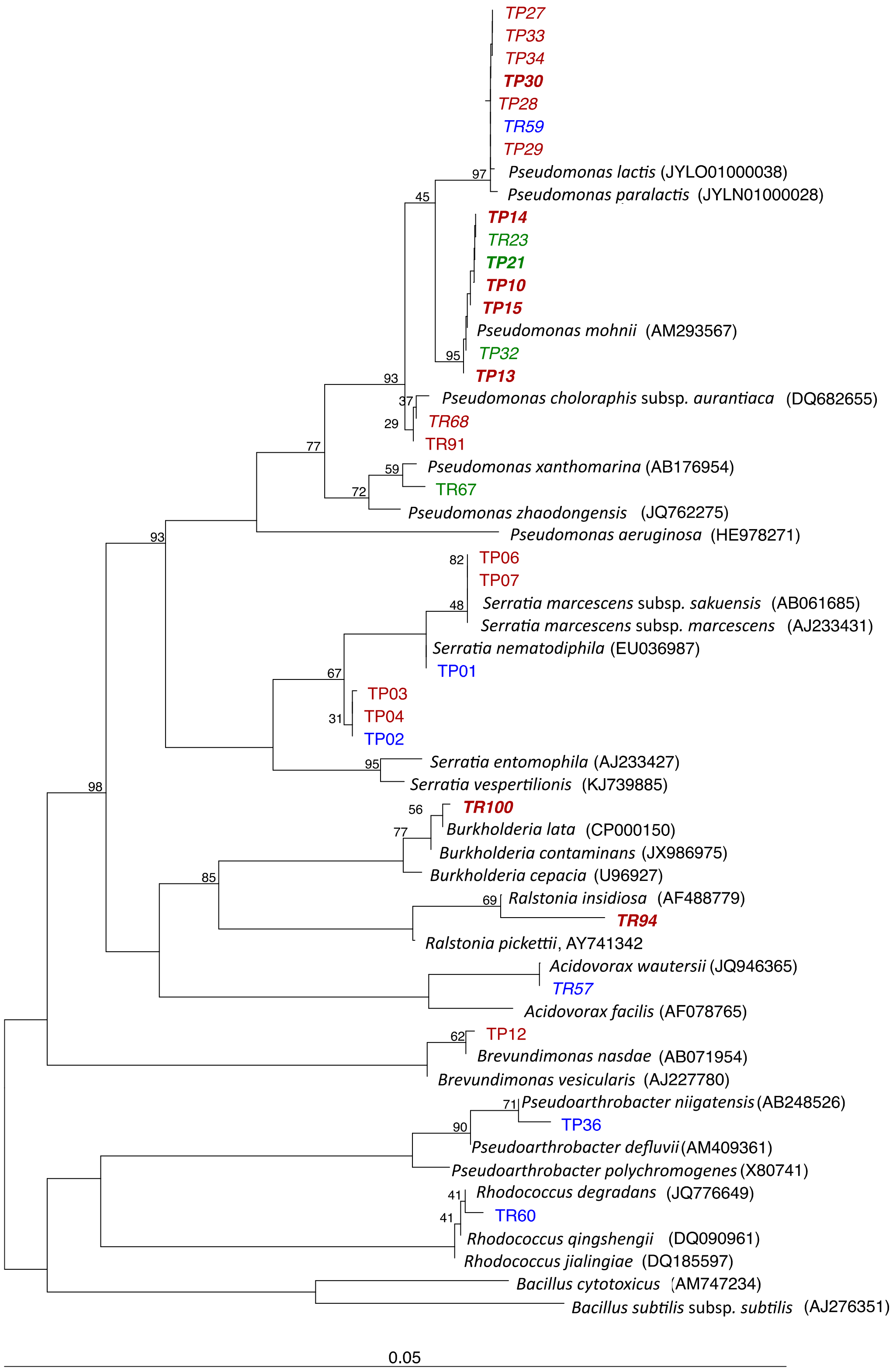

Supplementary Figure S6a. Evolutionary relationships of the *Proteobacteria* and *Actinobacteria* isolates obtained in this study.

The evolutionary history based on 16S rRNA gene sequences was inferred using the Neighbor-Joining method as described in the Methods section. The optimal tree is shown. The percentage of replicate trees in which the associated taxa clustered together in the bootstrap test (1000 replicates) are shown next to the branches. The tree is drawn to scale, with branch lengths in the same units as those of the evolutionary distances used to infer the phylogenetic tree. This analysis involved 58 nucleotide sequences. All ambiguous positions were removed for each sequence pair (pairwise deletion option). Isolates from water (blue), sediment (red) or forest soil (green) are in italics if they were *merA* positive. The highly resistant strains (resistant to ≥ 30 mg L^{-1} $HgCl_2$) are highlighted in bold. Strain code: TP if isolated from Tarapacá samples; TR if isolated from Taraira samples.

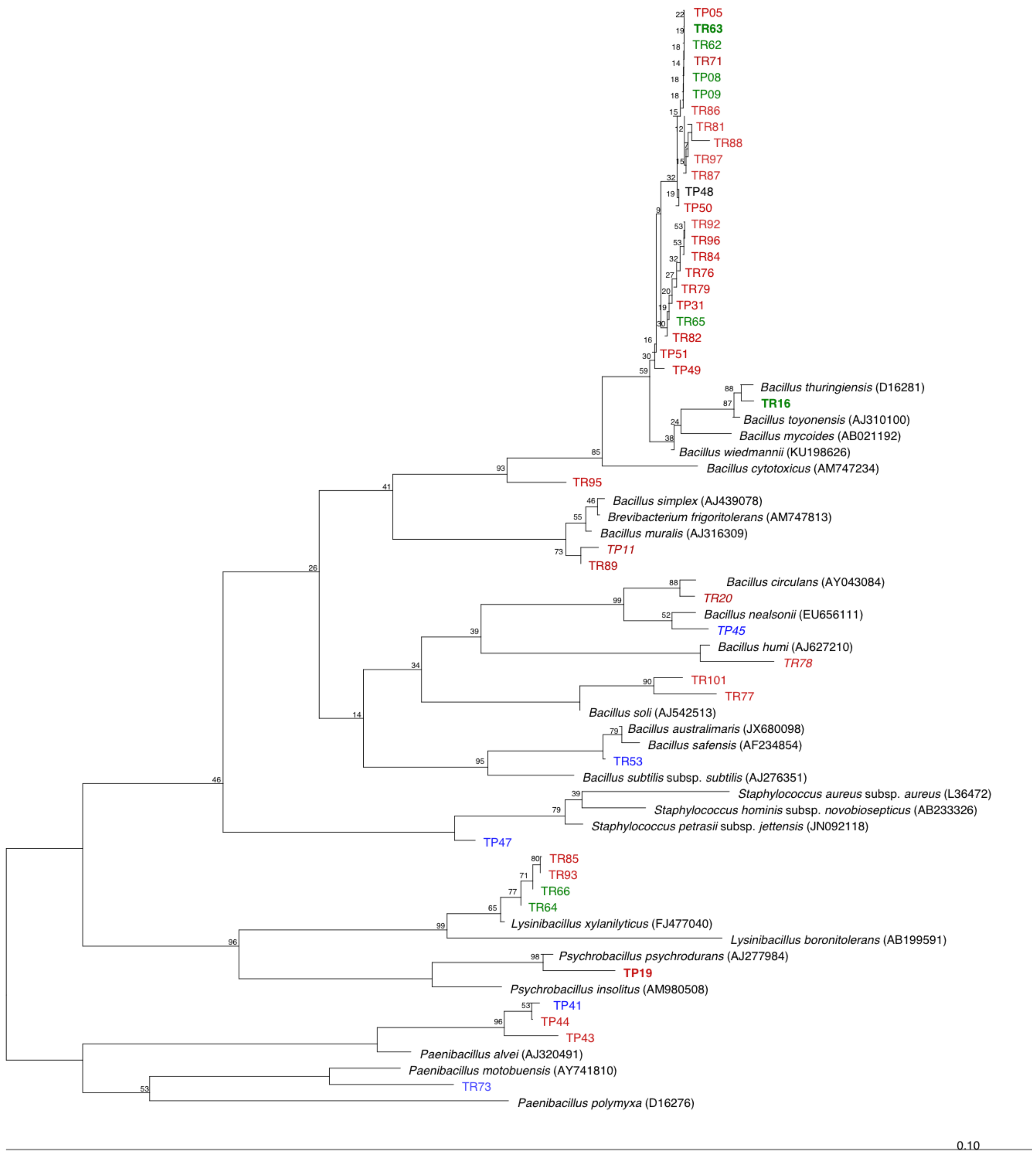

Supplementary Figure S6b. Evolutionary relationships of the *Firmicutes* isolates obtained in this study.

The evolutionary history based on 16S rRNA gene sequences was inferred using the Neighbor-Joining method as described in the Methods section. The optimal tree is shown. The percentage of replicate trees in which the associated taxa clustered together in the bootstrap test (1000 replicates) are shown next to the branches. The tree is drawn to scale, with branch lengths in the same units as those of the evolutionary distances used to infer the phylogenetic tree. This analysis involved 68 nucleotide sequences. All ambiguous positions were removed for each sequence pair (pairwise deletion option). Isolates from water (blue), sediment (red) or forest soil (green) are in italics if they were *merA* positive. The highly resistant strains (resistant to ≥ 30 mg L^{-1} $HgCl_2$) are highlighted in bold. Strain code: TP if isolated from Tarapacá samples; TR if isolated from Taraira samples.

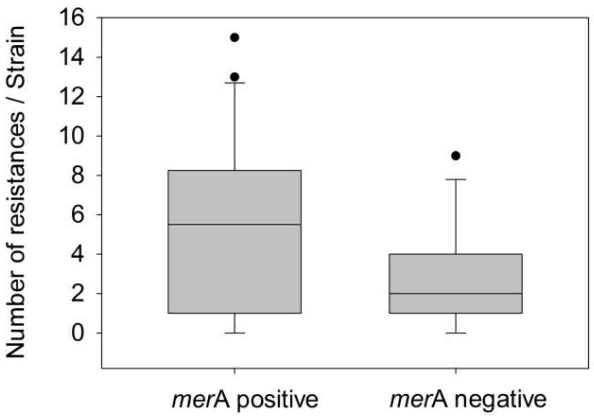

Supplementary Figure S7. Analysis of the presence of the mercury resistance *merA* gene and antibiotic resistances in the isolates. The data compare the number of antibiotic resistances detected in strains having or not the *merA* gene. A one-way ANOVA analysis showed the data were significantly different (p=0.041).

Supplementary Table S1A. Description of the sampling sites and samples in Tarapacá

Location	Site	Sample type	Sample Code	Description
Tarapacá	Tipisca lake	Water column	TL-W-1	Collected at 25% of the total width from the shore (Supplementary Fig.S1)
			TL-W-2	Collected in the middle of the waterbed
			TL-W-3	Collected at 75% of the total width from the shore
		Surface sediment	TL-SS-1	Collected at 25% of the total width from the shore. at 4.3 m depth.
			TL-SS-2	Collected in the middle of the waterbed. at 4.1 m depth.
			TL-SS-3	Collected at 75% of the total width from the shore at 4.2 m depth
		Interstitial sediment	TL-IS-1<br>TL-IS-2	Core collected at the shore. Two replicates
		Deep sediment core	TL-DS-1	Core collected at 4.15 m depth in the center of the lake. upper 1-7 cm sediment layer
			TL-DS-2	Core collected at 4.15 m depth in the center of the lake. 8-14 cm sediment layer.
			TL-DS-3	Core collected at 4.15 m depth in the center of the lake. 15-21 cm sediment layer.
		Forest soil	TL-FS-1	Composite samples from points A and B (Supplementary Fig.S3)
			TL-FS-2	Composite samples from points C and D (Supplementary Fig.S3)
			TL-FS-3	Sample collected at point E (Supplementary Fig.S3)
	Caño Pupuña	Water	CP-W-1<br>CP-W-2<br>CP-W-3	Collected along the riverbed. Three replicates
		Surface sediment	CP-SS-I<br>CP-SS-2<br>CP-SS-3	Collected along the riverbed. Three replicates
		Interstitial sediment	CP-IS-1<br>CP-IS-2	Core collected at the shore. Two replicates
		Deep sediment core	CP-DS-S	Sediment core collected in the centre of the river at 6.03 m depth. superficial layer.
			CP-DS-I	Sediment core collected in the centre of the river at 6.03 m depth. intermediate layer.
			CP-DS-D	Sediment core collected in the centre of the river at 6.03 m depth. deep layer.
		Forest soil	CP-FS-1	Composite samples from points A and B (Supplementary Fig.S3)
			CP-FS-2	Composite samples of points C and D (Supplementary Fig.S3)
			CP-FS-3	Sample collected at point E (Supplementary Fig.S3)
	Cotuhé river mouth	Water column	CoR-W	Collected in the middle of the waterbed
		Surface sediment	CoR-SS	Collected in the middle of the waterbed, at 3.9 m depth
	Putumayo river	Water column	PR-W	Collected in the middle of the waterbed
		Surface sediment	PR-SS	Collected in the middle of the waterbed, at 6.7 m depth

Supplementary Table S1B. Description of the sampling sites and samples in Taraira

Location	Site	Sample type	Code	Description
Taraira	Caño Amarillal upstream stretch	Water	CAu-W-0<br>CAu-W-1<br>CAu-W-2	Clear. shallow surface waters. 10-15 cm depth. Three replicates
		Surface sediment	CAu-SS-0<br>CAu-SS-1<br>CAu-SS-2	Sandy sediments with no macrophyte present. Three replicates
		Interstitial sediment	CAu-IS-0<br>CAu-IS-1	Collected at the shore. Two replicates.
		Forest soil (secondary. low forest cover)	CAu-FS-0	Composite samples from points A and B (Supp. Fig. S3)
			CAu-FS-1	Composite samples from points C and D (Supp. Fig. S3)
			CAu-FS-2	Sample collected at point E (Supplementary Fig. S3)
	Caño Amarillal downstream stretch	Water	CAd-W-0<br>CAd-W-1<br>CAd-W-2	Clear. shallow surface waters. 10-15 cm depth. Three replicates
		Surface sediment	CAd-SS-0<br>CAd-SS-1<br>CAd-SS-2	Sandy sediments with no macrophyte present. Three replicates.
		Interstitial sediment	CAd-IS	Collected at the shore, two replicates
		Forest soil	CAd-FS-0	Composite samples from points A and B (Supp. Fig. S3)
			CAd-FS-1	Composite samples from points C and D (Supp. Fig. S3)
			CAd-FS-2	Sample collected at point E (Supplementary Fig. S3)
	Caño Rojo upstream stretch	Water	CRu-W-0<br>CRu-W-1<br>CRu-W-2	Reddish. Shallow surface waters. Typical of the area. 10-15 cm depth. Three replicates
		Surface sediment	CRu-SS-0<br>CRu-SS-1<br>CRu-SS-2	Sandy sediments with no macrophyte present. Three replicates
		Interstitial sediment	CRu-IS-0<br>CRu-IS-2	Collected at the shore, two replicates.
		Forest soil (low forest cover)	CRu-FS-0	Composite samples from points A and B (Supp. Fig. S3)
			CRu-FS-1	Composite samples from points C and D ((Supp. Fig. S3)
			CRu-FS-2	Sample collected at point E (Supplementary Fig.S3)
	Caño Rojo downstream stretch	Water	CRd-W-0<br>CRd-W-1<br>CRd-W-2	Dark shallow water, 20 cm depth, macrophytes present
		Surface sediment	CRd-SS-0<br>CRd-SS-1<br>CRd-SS-2	Reddish, sandy sediments, three replicates
		Interstitial sediment	CRd-IS-0<br>CRd-IS-1	Collected at the shore, two replicates
		Forest soil	CRd-FS-0	Composite samples from points A and B (Supp. Fig. S3)
			CRd-FS-1	Composite samples from points C and D (Supp. Fig. S3)
			CRd-FS-2	Sample collected at point E (Supplementary Fig. S3)
		Deep sediment core	CRd-DS-S<br>CRd-DS-I<br>CRd-DS-D	Core collected in the middle of the waterbed, at 35 cm depth. Divided into surface (S), intermediate (I) and deep (D) layers.
	Caño Telecom	Water	CT-W-0<br>CT-W-1<br>CT-W-2	Dark waters collected at 20 cm depth. Macrophytes present. Three replicates.
		Surface sediment	CT-SS-0<br>CT-SS-1<br>CT-SS-2	Dark, sandy sediments, three replicates
		Interstitial sediment	CT-IS-1	Collected at the shore
		Forest soil	CT-FS-0	Composite samples from points A and B (Supp. Fig. S3)
			CT-FS-1	Composite samples from points C and D (Supp. Fig. S3)
			CT-FS-2	Sample collected at point E (Supplementary Fig. S3)

Table S2. Homology of the *merA* gene product amplified from some isolates against sequences in the databases.

Strain	Taxonomy[1]	Primer set				PCR product sequence length[2]	Protein sequence length	Closest MerA relative protein (refseq_protein db)	ID	Closest MerA relative protein (refseq_Env_protein db)	ID
		A1sn/A5n	merA Gram +	merA Gram -	merAFA1/R A5						
TP10	*Pseudomonas mohnii*	+		+	+	1023	341	*P. mohnii* (WP_198132017.1)<br>Other *Pseudomonas* (*P. aeruginosa*, *P. proteolytica*, *P. fulva*) and betaproteobacteria (*Acidovorax soli*, *Burkholderia multivorans*, *Enterobacter hormaechei*) species.	100<br>99.7	Hypothetical protein GOS_1986747 - marine metagenome (EDA51843.1). Surface water marine samples, collected along a voyage from Eastern North American coast to the Eastern Pacific Ocean.	94.7
TP13	*Pseudomonas mohnii*	+		+	+	1092	364				95
TP14	*Pseudomonas mohnii*	+		+	+	1077	359				94.9
TP15	*Pseudomonas mohnii*	+		+	+	1119	373				94.9
TP21	*Pseudomonas mohnii*			+	+	1059	353				94.6
TR23	*Pseudomonas mohnii*			+	+	1065	355				94.6
TP32	*Pseudomonas mohnii*	+			+	1059	353				94.9
TP27	*Pseudomonas paralactis*			+	+	486	162	*P. coronafaciens* (WP_122364113.1)<br><br>Other *Pseudomonas* species (*P. Syringae*, *P. aeruginosa*, *P. monteilii*, *P. fluorescens*).	100<br>98	Hypothetical protein LCGC14_0114400 - marine sediment metagenome (KKO01633.1) Loki's castle hydrothermal vent sediment	77.1
TP28	*Pseudomonas paralactis*			+	+	1080	360				80
TP29	*Pseudomonas paralactis*			+	+	1107	369				78.3
TP30	*Pseudomonas paralactis*			+	+	1680	560				74.4
TP33	*Pseudomonas paralactis*			+	+	651	217				76.5
TP34	*Pseudomonas paralactis*			+	+	1095	365				80
TR59	*Pseudomonas paralactis*			+	+	1080	360				80
TR68	*Pseudomonas chlororaphis*	+				186	62	*Aromatoleum toluvorans* (WP_169256443.1)	87.1	Mercuric ion reductase - mine drainage metagenome (EQD39500.1).	82.2
TR57	*Acidovorax wautersii*	+		+	+	1047	349	*Pseudomonas* spp. (WP_011666568.1)	100	Hypothetical protein GOS_1986747 - marine	100

								Other betaproteobacteria (*B. cenocepacia*, *A. baumannii*) and gammaproteobacteria (*P. monteilii*) species.	99	metagenome (EDA51843.1). Surface water marine samples, collected from Eastern North American coast to the Eastern Pacific Ocean.	
TR94	*Ralstonia insidiosa*			+	+	549	183	*R. pickettii* (WP_024973298.1)	95	Hypothetical protein GOS_1986747 - marine metagenome (EDA51843.1). Surface water marine samples, collected from Eastern North American coast to the Eastern Pacific Ocean.	89.6
TR100	*Burkholderia contaminans*	+				1677	559	*B. aenigmatica* (WP_059888155.1)	99.8		80.5
TP45	*Bacillus nealsonii*		+			474	158	*Streptococcus mitis* (WP_000630960.1) & *Dolosigranulum pigrum* (WP_181452786.1)	98.7	Mercuric reductase (MPM78518.1). Bioreactor metagenome	73.5
TR78	*Bacillus humi*		+			474	158	*Cytobacillus firmus* (WP_061794233.1)	98.7		73.2
TP11	*Bacillus frigoritolerans*		+			438	146	*diferent Bacillaceae species*	95 to 98		75.34
TR20*	*Bacillus circulans*		+								

[1] Table S3

[2] When the *merA* gene was amplified with different primer pairs, the longest fragment was selected for further analysis.

* No clean DNA sequence could be obtained from the amplified product.

Supplementary Table S3A. Total mercury (THg) and methyl mercury (MeHg) concentration in the sediment samples.

Location	Sample	Ecosystem	THg (mg Kg^{-1})	MeHg (mg Kg^{-1})
Taraira	CAu-SS-0	Surface sediment	*1.8851	0.02707
	CAu-SS-1		*10.4753	0.03396
	CAu-SS-2		*3.4903	0.01499
	CAd-SS-0		0.03872	0
	CAd-SS-1		0.01218	0
	CAd-SS-2		0.01239	0
	CRu-SS-0		0.06	0
	CRu-SS-1		0.09097	0
	CRu-SS-2		0.06356	0
	CRd-SS-0		*0.1208	0
	CRd-SS-1		*0.4668	0.00301
	CRd-SS-2		*0.1212	0
	CT-SS-0		0.01868	0
	CT-SS-1		0.02689	0
	CT-SS-2		0.03895	0
	CAu-IS-0	Interstitial sediment	*1.42	nd[1]
	CAu-IS-1		*6.47	
	CAd-IS		0.0096	
	CRu-IS-0		*0.099	
	CRu-IS-2		*0.094	
	CRd-IS-0		0.076	
	CRd-IS-1		*0.2446	
	CT-IS-1		0.0054	
	CRd-C-S	Deep sediment	*0.5608	
	CRd-C-I		*0.1162	
	CRd-C-D		*0.4187	
Tarapacá	TL-SS-1	Surface sediment	*0.0969	0.015
	TL-SS-2		0.033	0.007
	TL-SS-3		0.0857	0.003
	CP-SS-1		0.0783	0.002
	CP-SS-2		0.0644	0.002
	CP-SS-3		0.0677	0.003
	CoR-SS		*0.1013	0.004
	PR-SS		0.0618	0.003
	TL-IS-1	Interstitial sediment	0.085	nd
	TL-IS-2		0.076	
	TL-DS-1	Deep sediment	0.081	nd
	TL-DS-2		0.074	
	TL-DS-3		0.088	
	CP-IS-1	Interstitial sediment	0.079	nd
	CP-IS-2		0.087	
	CP-C-S	Deep sediment	*0.101	
	CP-C-I		0.093	
	CP-C-D		*0.095	

[1] Not determined

* Asterisks indicate values above the threshold limit.

Supplementary Table S3B. Total mercury (THg) and methyl mercury (MeHg) concentration in the water samples.

Sample	Location	THg (mg L^{-1})	MeHg (mg L^{-1})
CAu-W-0	Taraira	0.00027	<0.001[1]
CAu-W-1		0.00175	<0.001
CAu-W-2		0.00027	<0.001
CAd-W-0		* 0.10000	<0.001
CAd-W-1		0.00017	<0.001
CAd-W-2		0.00014	<0.001
CRu-W-0		0.0002	<0.001
CRu-W-1		0.00087	<0.001
CRu-W-2		0.0003	<0.001
CRd-W-0		0.0002	<0.001
CRd-W-1		0.00017	<0.001
CRd-W-2		0.00017	<0.001
CT-W-0		0.0002	<0.001
CT-W-1		*0.1000	<0.001
CT-W-2		*0.1000	<0.001
TL-W-1	Tarapacá	0.00017	0
TL-W-2		0.00015	0
TL-W-3		0.00017	0
CP-W-1		0.00014	0
CP-W-2		0.00014	0
CP-W-3		0.00014	0
CoR-W		0.00017	0
PR-W		0.00014	0

[*] Asterisks indicate values above the threshold limit
[1] 0.001 mg L^{-1} is the minimum detection threshold.

Supplementary Table S3C. Total mercury (THg) and methyl mercury (MeHg) concentration in the forest soil samples.

Sample	Location	THg (mg Kg^{-1})	MeHg (mg Kg^{-1})
CAu-FS.P0	Taraira	*13.2813	0.02356
CAu-FS.P1		*43.3488	0.0498
CAu-FS.P2		*1.6074	0.00456
CAd-FS-0		0.22414	0
CAd-FS-1		0.0719	0
CAd-FS-2		0.13982	0
CRu-FS-0		0.08844	0
CRu-FS-1		0.26683	0
CRu-FS-2		0.36115	0
CRd-FS-0		0.0871	0
CRd-FS-1		0.2986	0
CRd-FS-2		0.1249	0
CT-FS-0		0.05935	0
CT-FS-1		0.07289	0
CT-FS-2		0.01879	0
TL-FS-1	Tarapacá	0.1005	0.008
TL-FS-2		0.0836	0.007
TL-FS-3		0.0652	0.012
CP-FS-1		0.1411	0.009
CP-FS-2		0.1072	0.007
CP-FS-3		0.1107	0.003

[*] Asterisks indicate values above the threshold limit

Supplementary Table S4. Bacterial isolates affiliation, origin and characteristics.

OPU[1]	Isolate	Location	Site	Sample type	Closest relative	Type strain	Identity (%)	Accession number	Taxonomy
1	TP01	Tarapacá	Tipisca lake	water	*Serratia marcescens subsp. marcescens*	DSM 30121	99.85	AJ233431	Yersiniaceae (Enterobacterales, Gammaproteobacteria)
					Serratia marcescens subsp. Sakuensis	KRED	99.71	AB061685	
					Serratia nematodiphila	DZ0503SBS1	99.71	EU036987	
2*	TP06	Tarapacá	Tipisca lake	sediment	*Serratia marcescens subsp. Sakuensis*	KRED	99.86	AB061685	
					Serratia nematodiphila	DZ0503SBS1	99.72	EU036987	
					Serratia marcescens subsp. marcescens	DSM 30121	99.57	AJ233431	
	TP07				*Serratia marcescens subsp. Sakuensis*	KRED	99.87	AB061685	
					Serratia nematodiphila	DZ0503SBS1	99.74	EU036987	
					Serratia marcescens subsp. Marcescens	DSM 30121	99.61	AJ233431	
3*	TP02	Tarapacá	Caño Pupuña	water	*Serratia ficaria*	DSM 4569	98.77	AJ233428	
					Serratia entomophila	DSM 12358	98.50	AJ233427	
					Serratia vespertilionis	52	98.50	KJ739885	
	TP03		Tipisca lake	sediment	*Serratia ficaria*	DSM 4569	98.76	AJ233428	
					Serratia entomophila	DSM 12358	98.48	AJ233427	
					Serratia vespertilionis	52	98.48	KJ739885	
	TP04				*Serratia ficaria*	DSM 4569	98.73	AJ233428	
					Serratia entomophila	DSM 12358	98.44	AJ233427	
					Serratia vespertilionis	52	98.44	KJ739885	
4**	**TP27**	Tarapacá	Tipisca lake	sediment	*Pseudomonas paralactis*	DSM 29164	99.87	JYLN01000028	Pseudomonaceae (Pseudomonadales, Gammaproteobacteria)
					Pseudomonas lactis	DSM 29167	99.35	JYLO01000038	
	**TP28**				*Pseudomonas paralactis*	DSM 29164	99.85	JYLN01000028	
					Pseudomonas lactis	DSM 29167	99.55	JYLO01000038	
	**TP29**				*Pseudomonas paralactis*	DSM 29164	99.78	JYLN01000028	
					Pseudomonas lactis	DSM 29167	99.48	JYLO01000038	
	**TP30**		Caño Pupuña		*Pseudomonas paralactis*	DSM 29164	99.83	JYLN01000028	
					Pseudomonas lactis	DSM 29167	99.48	JYLO01000038	
	**TP33**				*Pseudomonas paralactis*	DSM 29164	99.87	JYLN01000028	
					Pseudomonas lactis	DSM 29167	99.36	JYLO01000038	

	**TP34**				*Pseudomonas paralactis*	DSM 29164	99.87	JYLN01000028	
					Pseudomonas lactis	DSM 29167	99.35	JYLO01000038	
	**TR59**	Taraira	Caño Amarillal	water	*Pseudomonas paralactis*	DSM 29164	99.85	JYLN01000028	
					Pseudomonas lactis	DSM 29167	99.54	JYLO01000038	
5**	TR68	Taraira	Caño Amarillal	sediment	*Pseudomonas chlororaphis subsp. Aurantiaca*	NCIB 10068	99.77	DQ682655	
	TR91						99.77		
6**	TP10	Tarapacá	Cotuhé river mouth		*Pseudomonas mohnii*	IpA-2	100	AM293567	
					Pseudomonas moorei	RW10	99.75	AM293566	
	TP13				*Pseudomonas mohnii*	IpA-2	99.93	AM293567	
					Pseudomonas moorei	RW10	99.85	AM293566	
	TP14				*Pseudomonas mohnii*	IpA-2	100	AM293567	
					Pseudomonas moorei	RW10	99.85	AM293566	
	TP15				*Pseudomonas mohnii*	IpA-2	100	AM293567	
					Pseudomonas moorei	RW10	99.73	AM293566	
	**TP21**		Tipisca lake	soil	*Pseudomonas mohnii*	IpA-2	100	AM293567	
					Pseudomonas moorei	RW10	99.74	AM293566	
	**TR23**	Taraira	Caño Rojo		*Pseudomonas mohnii*	IpA-2	100	AM293567	
					Pseudomonas moorei	RW10	99.74	AM293566	
	**TP32**	Tarapacá	Caño Pupuña		*Pseudomonas mohnii*	IpA-2	99.92	AM293567	
					Pseudomonas moorei	RW10	99.85	AM293566	
7	TR67	Taraira	Caño Amarillal		*Pseudomonas xanthomarina*	KMM 1447	99.14	AB176954	
					Pseudomonas zhaodongensis	NEAU-ST5-21	98.90	JQ762275	
8	TR57	Taraira	Caño Rojo	water	*Acidovorax wautersii*	NF1078	100	JQ946365	Comamonadaceae (Bulkholderiales, Betaproteobacteria)
9	**TR100**	Taraira	Caño Rojo	sediment	*Burkholderia contaminans*	LMG 23361	100	JX986975	Bulkholderiaceae (Bulkholderiales, Betaproteobacteria)
					Burkholderia lata	383		CP000150	
10	TR94	Taraira	Caño Rojo		*Ralstonia insidiosa*	AU2944	98.65	AF488779	
11	TP12	Tarapacá	Cotuhé river mouth		*Brevundimonas vesicularis*	LMG 2350	100	AJ227780	Caulobacteraceae (Caulobacterales, Alphaproteobacteria)
					Brevundimonas nasdae	GTC1043		AB071954	
12	TP36	Tarapacá		water	*Pseudarthrobacter defluvii*	AM409361	99.21	AM409361	

			Tipisca lake		*Pseudarthrobacter niigatensis*	LC4	99.08	AB248526	Micrococcaceae (Micrococcales, Actinobacteria)
13	**TR60**	Taraira	Caño Amarillal		*Rhodococcus jialingiae*	djl-6-2	100	DQ185597	Nocardiaceae (Actinomycetales, Actinobacteria)
					Rhodococcus qingshengii	djl-6		DQ090961	
					Rhodococcus degradans	CCM 4446		JQ776649	
14	**TP47**	Tarapacá	Putumayo river		*Staphylococcus petrasii subsp. jettensis*	SEQ110	99.21	JN092118	Staphylococcaceae (Bacillales, Firmicutes)
					Staphylococcus hominis subsp	GTC 1228	99.08	AB233326	
15*	TR64	Taraira	Caño Amarillal	soil	*Lysinibacillus xylanilyticus*	XDB9	99.77	FJ477040	
	TR66						99.76		
	TR85			sediment			99.86		
	TR93						99.73		
16	**TP19**	Tarapacá	Tipisca lake		*Psychrobacillus psychrodurans*	DSM 11713	99.24	AJ277984	
17**	TP05	Tarapacá	Tipisca lake		*Bacillus thuringiensis*	IAM 12077	99.40	D16281	
	**TP50**		Cotuhé river mouth		*Bacillus toyonensis*	CNCM I-1012/NCIB 40112		AJ310100	
18	TR95	Taraira	Caño Rojo		*Bacillus wiedmannii*	FSL W8-0169	98.19	KU198626	
					Bacillus mycoides	ATCC6462	98.05	AF013121	Bacillaceae (Bacillales, Firmicutes)
19	**TP31**	Tarapacá	Caño Pupuña		*Bacillus thuringiensis*	IAM 12077	99.54	D16281	
					Bacillus toyonensis	CNCM I-1012/NCIB 40112		AJ310100	
20**	TP49	Tarapacá	Cotuhé river mouth		*Bacillus toyonensis* / *Bacillus thuringiensis*	IAM 12077 / CNCM I-1012/NCIB 40112	99.41	AJ310100 / D16281	
	TR65	Taraira	Caño Amarillal	soil					
	TR79		Caño Rojo	sediment					
	**TR82**		Caño Amarillal						
	TR84				*Bacillus wiedmannii*	FSL W8-0169	99.54	KU198626	
	TR96		Caño Rojo				99.53		
21	TP51	Tarapacá			*Bacillus thuringiensis*	IAM 12077	99.63	D16281	

			Putumayo river		*Bacillus toyonensis*	CNCM I-1012/NCIB 40112	99.63	AJ310100	
22**	**TR76**	Taraira	Caño Amarillal		*Bacillus wiedmannii*	FSL W8-0169	99.87	KU198626	
	TR92								
23	TR88	Taraira	Caño Amarillal		*Bacillus thuringiensis*	IAM 12077	99.63	D16281	
					Bacillus toyonensis	CNCM I-1012/NCIB 40112		AJ310100	
24**	TP08	Tarapacá	Caño Pupuña	soil	*Bacillus toyonensis / Bacillus thuringiensis*	IAM 12077 / CNCM I-1012/NCIB 40112	99.55	AJ310100 / D16281	
	TP09								
	**TP48**			sediment					
	TR62	Taraira	Caño Amarillal	soil					
	TR63								
	TR71		Caño Telecom	sediment					
	TR86		Caño Amarillal						
	TR87								
	**TR81**								Bacillaceae (Bacillales, Firmicutes)
	TR97		Caño Rojo						
25	TR16	Taraira	Caño Rojo		*Bacillus toyonensis / Bacillus thuringiensis*	IAM 12077 / CNCM I-1012/NCIB 40112	100	AJ310100 / D16281	
26	TR53	Taraira	Caño Amarillal	water	*Bacillus safensis*	F0-036b	99.85	AF234854	
					Bacillus australimaris	MCC 1A05787	99.78	JX680098	
27*	TP11	Tarapacá	Cotuhé river mouth	sediment	*Bacillus frigoritolerans*	DSM 8801	99.48	AM747813	
	TR89	Taraira	Caño Amarillal		*Bacillus simplex*	DSM 1221T	99.34	AJ439078	
					Bacillus muralis	LMG 20238	99.34	AJ316309	
28*	**TR77**	Taraira	Caño Amarillal		*Bacillus soli*	LMG 21838	99.23	AJ542513	
	**TR101**		Caño Rojo				99.12		
29	**TR78**	Taraira	Caño Rojo		*Bacillus humi*	LMG 22167	99.40	AJ627210	

30	**TR20**	Taraira	Caño Rojo	water	*Bacillus circulans*	-	100	AY043084	
31	**TP45**	Tarapacá	Tipisca lake	water	*Bacillus circulans*	-	98.84	AY043084	
					Bacillus nealsonii	DSM 15077	98.59	EU656111	
32	TR73	Taraira	Caño Rojo	water	*Paenibacillus motobuensis*	MC10	97.72	NR_043153.1	Paenibacillaceae (Bacillales, Firmicutes)
33*	TP41	Tarapacá	Cotuhé river mouth	water	*Paenibacillus alvei*	DSM 29	98.94	AJ320491	
	TP44	Tarapacá		sediment			98.94		
	TP43	Tarapacá					98.80		

[1]OPU: Operational Phylogenetic Unit
in bold: isolated after liquid enrichment cycles.
*OPU: cluster of isolates with shared identity of at least 99.0 %
**OPU: cluster of isolates with shared identity of 100%

Supplementary Table S5. Yeast isolates and origin.

Strain	Location	Site	Closest relative	Acc. Number
TP35	Tarapacá	Tipisca Lake water	*Rhodotorula mucilaginosa*	KY611824.1
TP42		Caño Pupuña sediment	*Rhodotorula mucilaginosa*	MG270571.1
TR52	Taraira	Caño Amarillal water	*Rhodotorula mucilaginosa*	MF628270.1
TR55			*Yarrowia lipolytica*	MH459415.1
TR58		Caño Rojo water	*Aureobasidium pullulans*	MH134575.1
TR69		Caño Telecom sediment	*Rhodotorula mucilaginosa*	KY611824.1
TR70			*Cryptococcus laurentii*	KT899784.1
TR75			*Candida maltosa*	KY102187.1
TR80			*Rhodotorula mucilaginosa*	KY611824.1
TR90		Caño Amarillal sediment	*Rhodotorula mucilaginosa*	MF628270.1

Supplementary Table S6. Antibiotic resistance[1]. Presence of the *merA* gene and presence of plasmids in selected isolates.

			Fluoro-quinolones			Beta-lactams					Amino-glycosides				CP	TC	ML	GP	
**Strain**	***merA***	**plasmid**	**NOR10**	**OFX5**	**CIP5**	**AMX25**	**AM10**	**TIC75**	**IPM10**	**PIP100**	**KM30**	**S10**	**GM10**	**C30**	**CTX30**	**TC30**	**ER5**	**VA30**	**Total # of resistances**
TP1	−	−	S	S	S	R	R	nd	S	S	S	S	S	S	S[1]	S	R	nd	**3**
TP3	−	−	S	S	S	R	R	nd	S	S	S	S	S	S	S	S	S	nd	**2**
TP7	−	+	S	S	S	R	R	nd	S	S	S	S	S	S	R	S	S	nd	**3**
TP10	+	+	S	S	S	R	R	nd	S	S	S	S	S	S	S	S	R	R	**4**
TP11	+	−	S	S	S	S	S	nd	S	S	S	S	S	S	S	S	S	nd	**0**
TP13	+	−	S	S	R	R	R	nd	S	S	S	S	S	S	S	R	R	R	**6**
TP14	+	+	S	S	S	R	R	R	S	S	S	S	S	S	S	S	R	nd	**4**
TP15	+	+	S	S	S	S	S	nd	S	S	S	S	S	S	R	S	S	S	**1**
TP16	+	+	S	S	S	R	R	R	S	S	S	S	S	S	S	S	R	R	**5**
TP19	+	−	S	S	S	S	S	nd	S	S	S	S	S	S	R	S	S	S	**1**
TP20	+	−	nd	nd	S	S	nd	S	S	S	nd	S	S	S	nd	S	nd	nd	**0**
TP21	+	+	R	S	R	R	R	R	S	S	R	R	R	R	S	R	R	R	**12**
TP23	+	+	S	S	S	R	R	R	S	S	S	S	S	S	S	S	R	R	**5**
TP27	+	+	S	S	S	S	S	nd	S	S	R	R	S	S	S	S	S	S	**2**
TP28	+	−	S	S	S	R	R	nd	S	S	R	S	S	R	R	S	R	R	**7**
TP29	+	−	S	S	S	R	R	nd	S	S	R	S	S	R	R	S	R	R	**7**
TP30	+	+	S	R	R	R	R	R	S	S	R	R	R	R	S	R	R	R	**12**
TP31	−	−	S	S	S	R	R	nd	S	S	S	S	S	S	R	S	S	R	**4**
TP32	+	−	S	S	S	R	R	R	S	S	S	S	S	S	S	S	R	R	**5**
TP33	+	−	R	R	S	R	R	R	S	S	R	R	S	R	R	R	R	R	**12**
TP34	+	−	R	R	R	R	R	R	R	R	R	R	R	R	S	R	R	R	**15**
TP45	+	−	S	S	S	S	S	nd	S	S	S	S	S	S	R	S	S	S	**1**
TP47	−	+	S	S	S	S	S	nd	S	S	S	S	S	S	R	S	S	nd	**1**
TP51	−	−	S	nd	S	R	R	R	S	S	S	S	S	S	R	S	S	S	**4**
TR57	+	−	S	S	S	S	S	S	S	S	S	S	S	S	S	S	S	S	**0**
TR59	+	−	S	nd	S	R	R	R	S	S	R	S	S	S	S	S	R	R	**6**
TR62	−	+	S	S	S	S	S	nd	S	S	S	S	S	S	R	S	S	S	**1**
TR63	−	+	S	S	S	S	S	nd	S	S	S	S	S	S	R	S	S	S	**1**
TR68	+	−	S	nd	S	R	R	R	S	S	S	S	S	S	R	S	R	R	**6**
TR71	−	+	S	S	S	S	S	nd	S	S	S	R	S	S	S	S	S	S	**1**
TR78	+	−	S	S	S	S	S	S	S	S	S	S	S	S	S	S	S	S	**0**
TR79	−	−	S	S	S	S	S	nd	S	S	S	S	S	S	S	S	S	S	**0**
TR89	−	−	S	S	S	R	R	R	S	R	R	R	R	S	S	R	R	S	**9**
TR91	−	−	S	nd	S	R	R	R	S	S	S	S	S	S	R	S	R	R	**6**
TR94	+	−	R	R	R	R	R	R	R	R	R	S	R	S	R	S	R	R	**13**
TR100	+	+	S	S	S	R	R	nd	S	S	R	R	R	S	S	S	R	R	**7**
TR101	−	−	S	S	S	S	S	S	S	S	S	S	S	S	S	S	S	S	**0**

[1] The concentration (in µg/ml) of the antibiotics assayed is indicated with a figure after the abbreviation. Fluoroquinolones: CIP, ciprofloxacin; NOR, norfloxacin; OFX, ofloxacin. Beta-lactams: IPM, imipenem; PIP, piperacillin; AMX, amoxicillin; AM, ampicillin; TIC, ticarcillin. Aminoglycosides: KM, kanamycin; GM, gentamicin; S, streptomycin. Macrolides (ML): ER, erythromycin. Glycopeptides (GP): VA, vancomycin. Cephalosporine (CP): CTX, cefotaxime. Tetracyclines (TC): TC, tetracycline. C, chloramphenicol;

Supplementary Table S7. Reported maximum MIC values in previously published mercury resistant bacteria

Strains	Hg resistance: $HgCl_2$ MIC (mg L^{-1})	Ecosystem	*merA* / Comments	Reference
Pseudomonas putida SP1	81.456	Seawater	The *mer* operon was located on Tn5041 on the chromosome	(Zhang et al., 2012)
Bacillus megaterium MB1	2	Marine sediments	Tn21-type *mer* operon in one copy on the chromosome	(Huang et al., 1999)
Aeromonas media<br>*Bacillus* spp.<br>*Citrobacter freundii*<br>*Vibrio* spp<br>*Serratia marcescens*	13.4<br>0.68 – 6.8<br>13.62<br>1.4 – 13.4<br>6.81	Estuarine sediments	*Aeromonas media* (+)<br>*Citrobacter freundii* (+)<br>*Bacillus, Vibrio, Serratia* (–)	(Figueiredo et al., 2016)
Bacillus sp.; *Enterococcus* sp.<br>*Bacillus* spp.<br>*Halobacillus* spp., *Virgibacillus* sp.<br>*Pseudomonas* sp.<br>*Psychrobacter* sp.; *Kocuria* sp.<br>*Brachybacterium* spp.<br>Halomonas spp.	20.36<br>≥ 27.152<br>≥ 27.152<br>≥ 27.152<br>≥ 27.152<br>≥ 27.152<br>≥ 27.152	Marine sponges	The *merA* gene was detected in all strains except *Enteroccus* sp., *Virgibacillus* sp., *Psychrobacter* sp., 2 of the 8 *Bacillus* sp. strains, and 1 of the 2 *Halobacillus* sp. strains.	(Santos-Gandelman et al., 2014)
Bacillus cereus<br>*Lysinibacillus* sp.<br>*Bacillus* sp.<br>*Kocuria rosea*<br>*Microbacterium oxydans*<br>*Serratia marcescens*<br>*Ochrobactrum* sp.	8.15<br>16.3<br>27.15<br>5.4<br>27.15<br>16.3<br>16.3	Soils and sediments	The resistance mechanism was based on extracellular bioaccumulation through EPS	(François et al., 2012)
Pseudomonas spp.<br>*Acinetobacter* spp.<br>*Proteus* spp.<br>*Serratia marcescens*<br>*Micrococcus* sp.<br>*Klebsiella* spp.<br>*Staphylococcus*<br>*Escherichia coli*	25–100<br>6.25–25<br>25–50<br>12.5–50<br>6.25<br>6.25–12.5<br>6.25–12.5	Sediments and water	Not searched. Plasmids were detected in several *Serratia marcescens* and *Pseudomonas* strains	(Kargar et al., 2012)

Bacillus spp.	6.25–12.5<br>6.25–12.5			
Enterobacter spp.<br>*Pseudomonas* spp.	67.88 - 108.6<br>67.88 - 249.8	Soils	*merA* gene detected in 4 out of 6 *Pseudomonas* strains, and 1 of the 2 *Enterobacter* isolates. The strains with *merA* were capable of mercury reduction.	(Giovanella et al., 2016)
Psychrobacter spp.<br>*Pseudomonas* spp.	13.5 – 27.1<br>20.4	Brackish lagoon sediment	Not searched. The two *Pseudomonas* strains and one *Psychrobacter* strain could reduce mercury.	(Pepi et al., 2011)
Pseudomonas spp.<br>*Enterobacter* spp.<br>*Serratia* spp.<br>*Providencia alcalifaciens.*<br>*Bacillus cereus*	0.73 – 3.12<br>1.11 – 1.49<br>1.49 – 2.5<br>2.5<br>1.36 – 2.61	Soils after mining sewage treatments	*merA* gene detected in one highly resistant *Pseudomonas* strain	(Cabral et al., 2013)
TP10 *Pseudomonas mohnii,*<br>TP11 *Bacillus frigoritolerans,*<br>TP13 *Pseudomonas mohnii,*<br>TP14 *Pseudomonas mohnii,*<br>TP15 *Pseudomonas* sp., TP16 *Bacillus* sp.<br>TP30 *Pseudomonas* sp., TR63 *Bacillus* sp.<br>TR94 *Ralstonia insidiosa,*<br>TR100 *Burkholderia contaminans*	60	Lake and river sediments, forest soils	TP10, TP11, TP13, TP14, TP15, TP16, TP30, TR94, TR 100 (+)<br>TR63 (–)	This Study

References

Cabral, L, Giovanella, P, Gianello, C, Bento, FM, Andreazza, R, and Camargo, FA (2013) Isolation and characterization of bacteria from mercury contaminated sites in Rio Grande do Sul, Brazil, and assessment of methylmercury removal capability of a Pseudomonas putida V1 strain, *Biodegradation* **24**: 319-331. https://doi.org/10.1007/s10532-012-9588-z

Figueiredo, NL, Canário, J, O'Driscoll, NJ, Duarte, A, and Carvalho, C (2016) Aerobic Mercury-resistant bacteria alter Mercury speciation and retention in the Tagus Estuary (Portugal), *Ecotoxicol Environ Saf* **124**: 60-67. https://doi.org/10.1016/j.ecoenv.2015.10.001

François, F, Lombard, C, Guigner, J-M, Soreau, P, Brian-Jaisson, F, Martino, G, Vandervennet, M, Garcia, D, Molinier,AL, Pignol,D, Peduzzi, J, Zirah,S and Rebuffat, S (2012) Isolation and characterization of environmental bacteria capable of extracellular biosorption of mercury, *Appl Environ Microbiol* **78**: 1097-1106. https://doi.org/10.1128/AEM.06522-11

Giovanella, P, Cabral, L, Bento, FM, Gianello, C, and Camargo, FA (2016) Mercury (II) removal by resistant bacterial isolates and mercuric (II) reductase activity in a new strain of Pseudomonas sp. B50A, *N Biotechnol* **33**: 216-223. https://doi.org/10.1016/j.nbt.2015.05.006

Huang, CC, Narita M Fau - Yamagata, T, Yamagata T Fau - Itoh, Y, Itoh Y Fau - Endo, G, and Endo, G (1999) Structure analysis of a class II transposon encoding the mercury resistance of the Gram-positive Bacterium bacillus megaterium MB1, a strain isolated from minamata bay, Japan, *Gene* **234**: 361-369.

Kargar, M, Jahromi, M, Najafian, M, Khajeaian, P, Nahavandi, R, Jahromi, S, and Firoozinia, M (2012) Identification and molecular analysis of mercury resistant bacteria in Kor River, Iran., *Afr J Biotechnol* **11**: 6710-6717. https://doi.org/10.5897/AJB11.3394

Pepi, M, Gaggi, C, Bernardini, E, Focardi, S, Lobianco, A, Ruta, M, Nicolardia, V, Volterrania, M, Gasperini, S, Trinchera, G, Renzi, P, Gabellini, M and Focardia, SE (2011) Mercury-resistant bacterial strains Pseudomonas and Psychrobacter spp. isolated from sediments of Orbetello Lagoon (Italy) and their possible use in bioremediation processes, *Int Biodeterior Biodegradation* **65**: 85-91. https://doi.org/10.1016/j.ibiod.2010.09.006

Santos-Gandelman, JF, Giambiagi-deMarval, M, Muricy, G, Barkay, T, and Laport, MS (2014) Mercury and methylmercury detoxification potential by sponge-associated bacteria, *Antonie Van Leeuwenhoek* **106**: 585-590. https://doi.org/10.1007/s10482-014-0224-2

Zhang, W, Chen, L, and Liu, D (2012) Characterization of a marine-isolated mercury-resistant Pseudomonas putida strain SP1 and its potential application in marine mercury reduction, *Appl Microbiol Biotechnol* **93**: 1305-1314. 10.1007/s00253-011-3454-5
